# Photogeneration of Spin Quintet Triplet–Triplet Excitations in DNA-Assembled Pentacene Stacks

**DOI:** 10.1021/jacs.2c13743

**Published:** 2023-02-24

**Authors:** Sarah R. E. Orsborne, Jeffrey Gorman, Leah R. Weiss, Akshay Sridhar, Naitik A. Panjwani, Giorgio Divitini, Peter Budden, David Palecek, Seán T. J. Ryan, Akshay Rao, Rosana Collepardo-Guevara, Afaf H. El-Sagheer, Tom Brown, Jan Behrends, Richard H. Friend, Florian Auras

**Affiliations:** † Cavendish Laboratory, Department of Physics, University of Cambridge, CB3 0HE Cambridge, U.K.; ‡ Pritzker School of Molecular Engineering, University of Chicago, Chicago, Illinois 60637, United States; § Department of Applied Physics, Science for Life Laboratory, KTH Royal Institute of Technology, 17121 Solna, Sweden; ∥ Berlin Joint EPR Laboratory, Fachbereich Physik, Freie Universität Berlin, 14195 Berlin, Germany; ⊥ Department of Materials Science & Metallurgy, University of Cambridge, CB3 0FS Cambridge, U.K.; # Yusuf Hamied Department of Chemistry, University of Cambridge, Cambridge CB2 1EW, U.K.; ¶ Department of Chemistry, University of Oxford, OX1 3TA Oxford, U.K.; ∇ Department of Science and Mathematics, Faculty of Petroleum and Mining Engineering, Suez University, Suez 43721, Egypt

## Abstract

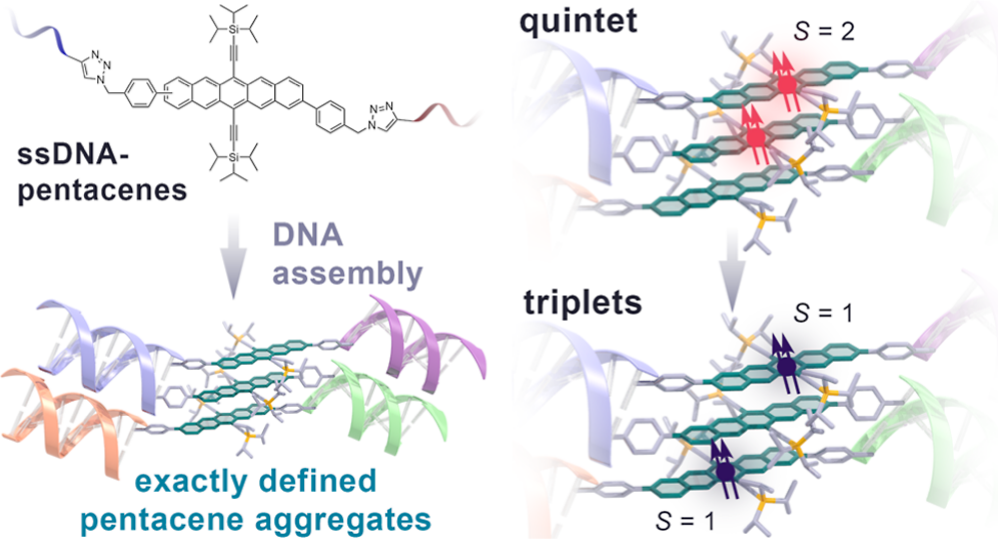

Singlet fission (SF), an exciton-doubling process observed in certain molecular semiconductors where two triplet excitons are generated from one singlet exciton, requires correctly tuned intermolecular coupling to allow separation of the two triplets to different molecular units. We explore this using DNA-encoded assembly of SF-capable pentacenes into discrete π-stacked constructs of defined size and geometry. Precise structural control is achieved via a combination of the DNA duplex formation between complementary single-stranded DNA and the local molecular geometry that directs the SF chromophores into a stable and predictable slip-stacked configuration, as confirmed by molecular dynamics (MD) modeling. Transient electron spin resonance spectroscopy revealed that within these DNA-assembled pentacene stacks, SF evolves via a bound triplet pair quintet state, which subsequently converts into free triplets. SF evolution via a long-lived quintet state sets specific requirements on intermolecular coupling, rendering the quintet spectrum and its zero-field-splitting parameters highly sensitive to intermolecular geometry. We have found that the experimental spectra and zero-field-splitting parameters are consistent with a slight systematic strain relative to the MD-optimized geometry. Thus, the transient electron spin resonance analysis is a powerful tool to test and refine the MD-derived structure models. DNA-encoded assembly of coupled semiconductor molecules allows controlled construction of electronically functional structures, but brings with it significant dynamic and polar disorders. Our findings here of efficient SF through quintet states demonstrate that these conditions still allow efficient and controlled semiconductor operation and point toward future opportunities for constructing functional optoelectronic systems.

## Introduction

Singlet fission (SF) is a spin-conserving process in which one singlet exciton evolves to form two triplet excitons on neighboring molecules.^1,2^ Since this exciton doubling process allows the quantum yield of solar cells to exceed 100%, SF has the potential to overcome the thermodynamic limit for single-junction solar cells.^3,4^

SF in acenes is understood to proceed via a triplet pair state, ^1^(T_1_T_1_), formed on adjacent molecules, which can subsequently dissociate into pairs of free triplets.^5,6^ Most studies have focused on pentacenes where the evolution to free triplet pairs can proceed very rapidly,^7,8^ and tetracenes where there is evidence for a stronger role for the intermediate^1^(T_1_T_1_) state.^9,10^ Since the SF process inherently requires at least two electronically coupled chromophores, its kinetics and triplet yield are highly sensitive to intermolecular geometry and connectivity.^11^

Transient electron spin resonance (trESR) spectroscopy has been employed to track the evolution of spin states, revealing the formation of a spin quintet intermediate, ^5^(T_1_T_1_), in some SF systems.^12−14^ The quintet ESR signature provides direct experimental evidence for a bound triplet pair state, and the analysis of its ESR spectrum and zero-field-splitting (ZFS) parameters can provide information about the spatial extent and geometry of this state.^15^

For high-spin quintet intermediates to persist on the timescale of an ESR experiment without rapidly dephasing, SF chromophores are typically linked to form well-defined dimers or oligomers.^16,17^ While this covalent approach does offer tunability and rigid control over geometry, it is synthetically challenging to extend the number of coupled SF chromophores.^18−20^ In an alternative approach, the doping of pentacene in a *p*-terphenyl host matrix has been used to generate pentacene nanoclusters.^21^ TrESR analysis identified two distinct quintet states that were attributed to parallel and herringbone intermolecular geometries. Only the parallel configuration was found to promote the separation of the triplet pair state into free triplets. Recently, molecular engineering to control the packing in bis(tricyclohexylsilylethynyl)tetracene single crystals has been used to achieve high-spin ^5^(TT) multiexcitons.^22^ However, even these well-engineered materials cannot provide exact control over the aggregate size and geometry, and alternative, deterministic approaches are needed to achieve this goal.

DNA has been utilized to assemble molecular semiconductors into pre-programed architectures.^23,24^ Here, the exact recognition of complementary base sequences in combination with the rigid and predictable helical structure of double-stranded DNA (dsDNA) is used to generate scaffolds that position appended chromophores into precisely defined geometries.^25−28^

Here, we report the DNA-encoded assembly of pentacene dimers, trimers, and pentamers. We have constructed precisely defined slip-stacked pentacene aggregates by employing a hierarchy of interactions: the DNA duplex formation controls the overall aggregate size, while hydrophilic–hydrophobic interactions and the local molecular geometry direct the chromophores into a well-defined intermolecular arrangement. This approach allows us to position SF chromophores in a π-stacked configuration with through-space electronic interactions as found in crystalline SF materials, but with the exact size control of covalently interconnected oligomers.

By using fast transient optical spectroscopy, we observe fast SF where the SF rate increases with the stack size. At cryogenic temperatures, trESR spectroscopy reveals that SF proceeds via a bound triplet pair quintet state with a well-defined intermolecular geometry in all DNA/pentacene constructs, which subsequently converts into free triplets. Thus, SF acts as a highly specific test to reveal intermolecular organization.

Our DNA assembly strategy provides an opportunity to realize efficient and controlled semiconductor operation even in highly polar environments and points toward future opportunities for constructing functional optoelectronic systems.

## Results and Discussion

**Figure 1:**
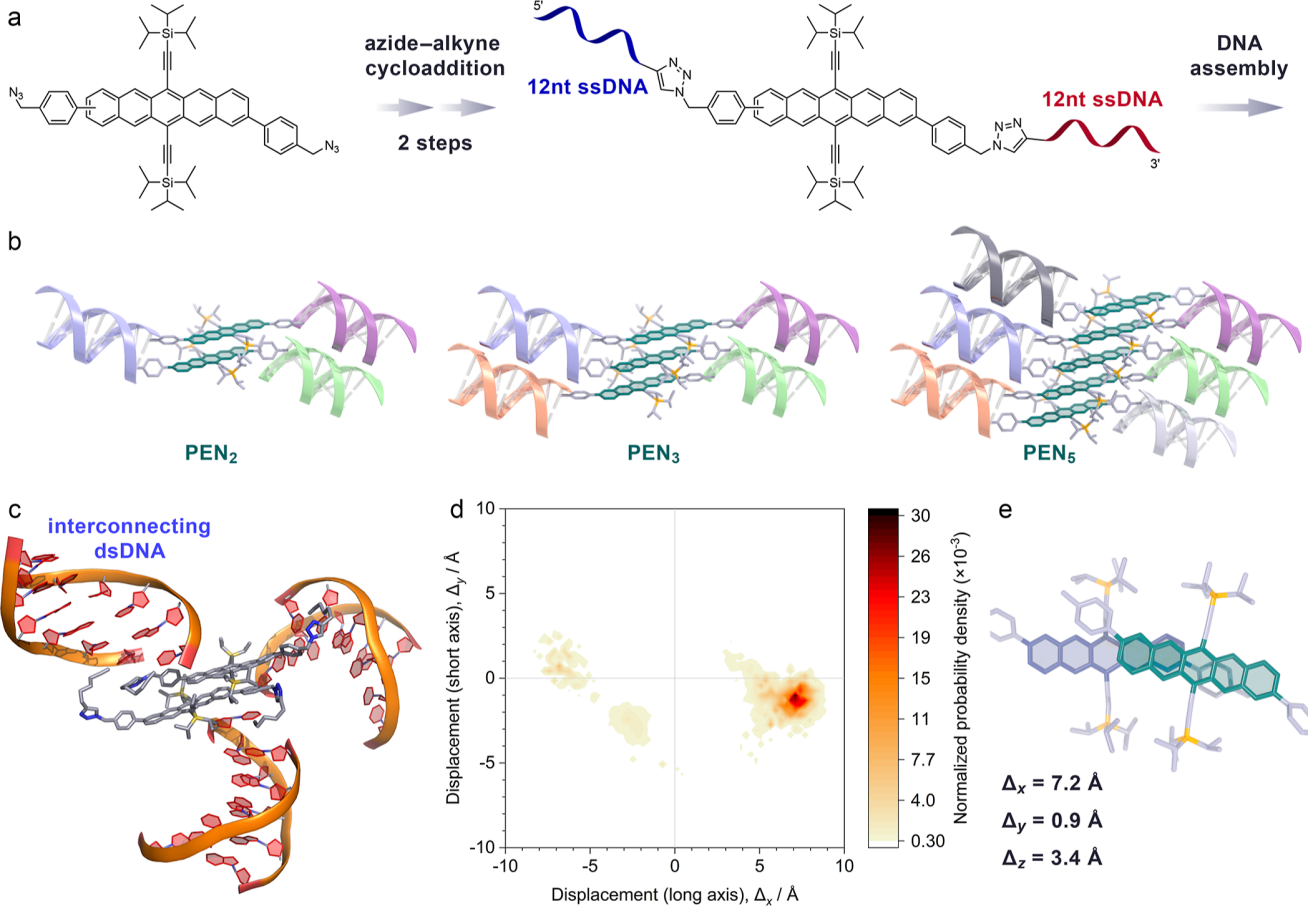
Assembly and modeling of the pentacene/DNA constructs. (a) DNA-functionalized pentacenes are synthesized via Cu(I)-catalyzed azide–alkyne cycloaddition. Two 12 nucleotide (nt) single-stranded DNAs (ssDNAs) of different base sequences are attached sequentially while preserving the directionality of the DNA. This process is repeated to generate a library of pentacenes with complementary sequences. Pentacene dimers, trimers, and pentamers are generated by selecting the desired components from this library, followed by hybridization to form rigid dsDNAs. (b) Schematic illustration of the DNA-linked pentacene constructs (**PEN**_***n***_) that consist of *n* pentacenes, interconnected by (*n* – 1) dsDNA and two terminating dsDNAs. Color coding represents complementary base sequences. (c) Simulated MD-optimized geometry of **PEN**_**2**_. The DNA arranges radially around the hydrophobic pentacenes. (d) Normalized probability density map showing the lateral offset along the long (*x*) and short (*y*) molecular axes of the pentacenes in **PEN**_**2**_. Driven by hydrophilic–hydrophobic interactions and guided by the local geometry due to the bulky TIPS-ethynyl substituents, the pentacenes are positioned in a well-defined arrangement. (e) Cut-out of the optimized **PEN**_**2**_ geometry, showing the arrangement of the pentacenes with considerable offset along the long molecular axis. The pentacenes are closely π-stacked with an average distance of 3.4 Å.

In order to link pentacenes to DNA, we employed copper(I)-catalyzed azide–alkyne cycloaddition chemistry. While more oxidation-stable semiconductors such as perylene diimides can be introduced directly in the solid-phase oligonucleotide synthesis,^28−30^ the required harsh conditions are not compatible with pentacenes. Instead, we installed azide groups along the pentacene long molecular axis, which allow the facile coupling to alkyne-modified DNA (Figure 1a). Two bulky triisopropylsilylethynyl (TIPS-ethynyl) groups on the 6th and 13th positions of the pentacene core increase the chemical stability and generate a molecular docking site that guides neighboring pentacenes into a well-defined geometry, as discussed below.^31,32^

We have found that ssDNA-functionalized pentacenes can self-assemble spontaneously into dimers due to amphiphilic contrast (Supporting Information, Section E). This confirms that the structures we have selected are capable of arranging the pentacene moieties to allow an electronic contact. However, this self-assembly is limited to dimers and does not allow assembly of more extended pentacene stacks by design. The full potential of DNA lies in its duplex formation with a complementary base sequence. By installing two ssDNAs of different base sequences on either side of the pentacenes, we generate handles for attaching further building blocks. Pentacene stacks can thus be assembled with exact size control via DNA hybridization (Figure 1b).

A major challenge with copper(I)-catalyzed azide–alkyne cycloaddition is to attach two different ssDNA strands while maintaining the directionality of the DNA. We solved this by first coupling the pentacenes to the 5′ end of resin-bound ssDNA and then attaching 3′ alkyne-dU modified DNA to the remaining unreacted azide group (see Supporting Information, Section B for details). Repeating this process with complementary base sequences, followed by hybridization to form dsDNA yields the desired DNA-linked pentacene stacks. We focus in this study on the dimers, trimers, and pentamers (denoted as **PEN**_***n***_; *n* = 2,3,5; see Figure 1b).

The electronic coupling within the stacks depends on the distances and orientation of the pentacenes relative to each other. The DNA scaffold dictates the stack size and provides coarse positioning of the pentacenes. Hydrophobic–hydrophilic differentiation between the non-polar pentacenes and the surrounding highly polar aqueous environment forces the pentacenes into close proximity. On a sub-nanometer scale, the local geometry of the aromatic pentacene cores with their very bulky TIPS-ethynyl substituents guides adjacent pentacenes into a coplanar configuration with substantial lateral offset.

As the pentacene intermolecular geometry within the **PEN**_***n***_ constructs cannot be resolved directly via electron microscopy or scattering techniques, we employed atomistic molecular dynamics (MD) simulations in combination with a well-tempered metadynamics algorithm for enhanced sampling (see Supporting Information, Section D for details).^33,34^ We have demonstrated previously that this technique can be adapted to generate accurate structure models of DNA-assembled molecular semiconductors.^28^ The MD-generated **PEN**_***n***_ geometries were found to be consistent with the analysis of the spin quintet spectra observed in trESR experiments, when a slight systematic strain was added (see below).

MD simulations were initialized with non-aggregated pentacenes and allowed both the DNA and semiconductors to sample a wide range of configurations including the dehybridization of the dsDNA.

**PEN**_**2**_ converges to a closely stacked pentacene dimer with the interconnecting and terminal dsDNA extending radially to minimize the interface between the hydrophobic semiconductors and the surrounding aqueous medium (Figure 1c). This DNA shell provides excellent steric shielding, confining electronic interactions to the individual pentacene stacks with minimal crosstalk across multiple pentacene/DNA constructs.

In the optimized geometry, the pentacenes form a cofacially stacked dimer with an average stacking distance (Δ_*z*_) of 3.4 Å (Figure 1e). However, due to the bulky TIPS-ethynyl groups and steric constraints of the attached DNA, there is a substantial lateral offset of more than half the pentacene length and width along the long and short molecular axes, respectively.

Due to the inherent flexibility of the **PEN**_***n***_ constructs, thermal movement will lead to a distribution of configurations. In order to assess this range of possible pentacene arrangements, we computed a probability density (NPD) map. This shows the probability histogram of configurations with displacement along the long (Δ_*x*_) and short (Δ_*y*_) molecular axes and the stacking direction (Figure 1d and Supporting Information, Figure S1d). The NPD map reveals significant probability for configurations with displacements slightly shorter or longer than the optimized geometry.

The **PEN**_***n***_ constructs are interconnected by (*n* – 1) dsDNA plus two terminating dsDNAs. We find that due to the slip-stacked arrangement of the pentacenes, this does not lead to significant steric crowding between the dsDNA and does not introduce a limit to the number of pentacenes that can be assembled this way (Supporting Information, Figure S5).

For further structural characterization, we used cryogenic transmission electron microscopy to obtain an indication of the size of the pentacene/DNA constructs (Supporting Information, Figure S6).

**Figure 2:**
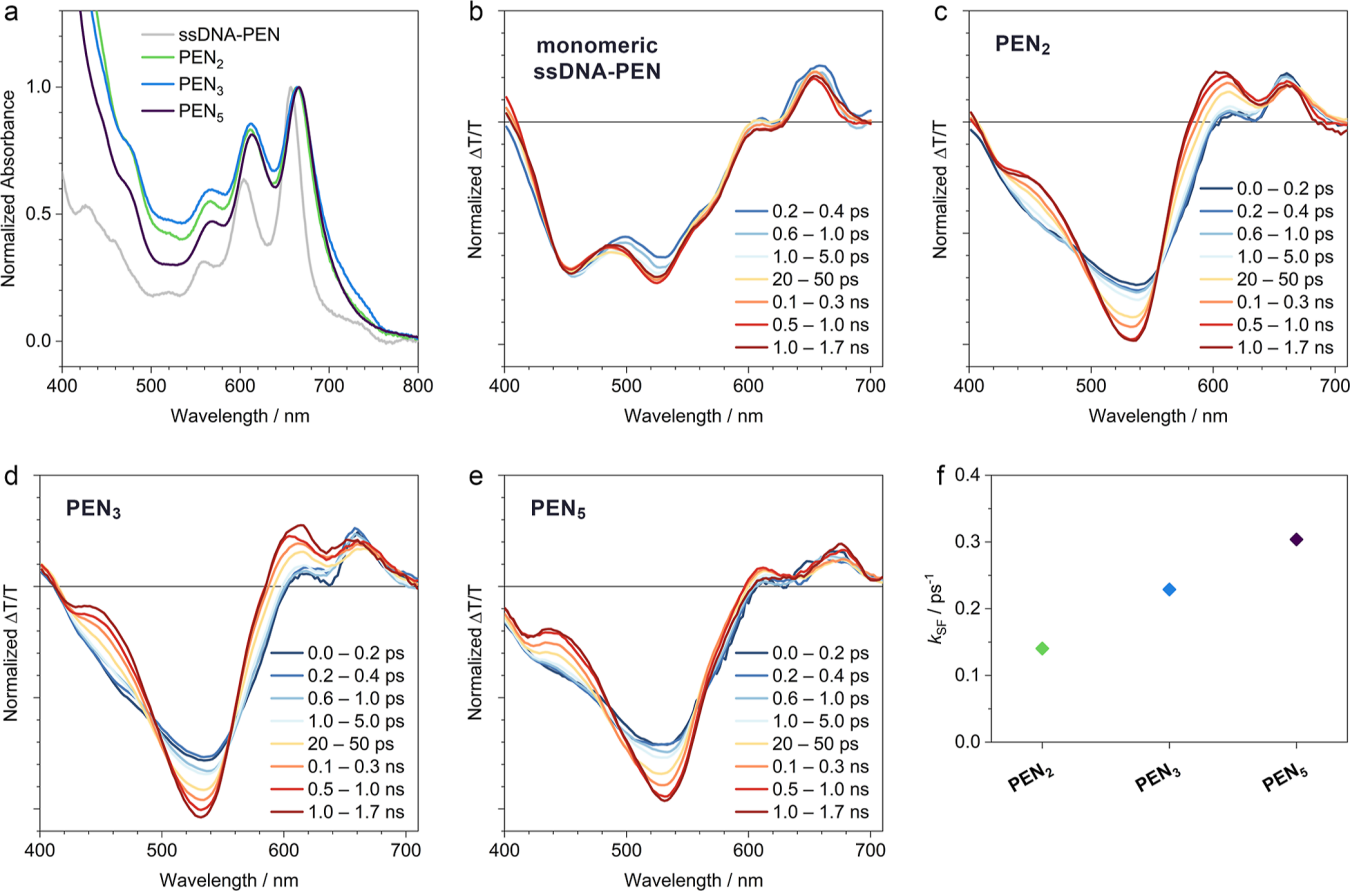
Spectroscopic characterization. The DNA-linked **PEN**_***n***_ are dissolved in phosphate-buffered saline with a pentacene concentration of 100 μM. The monomeric ssDNA-PEN is dissolved in a DMSO/buffer mixture (95:5 v/v) to suppress aggregation. (a) Steady-state absorption spectra of the DNA-linked pentacenes and the monomeric ssDNA-PEN. (b–e) Femto-/picosecond TA spectra of the samples, following photoexcitation at 600 nm (pump fluence 5 × 10^–5^ J cm^–2^). Spectra are normalized to their respective integrals. (f) SF rate constants derived from exponential fits of the deconvoluted spectra.

The DNA/pentacene constructs show the typical optical characteristics of pentacenes with absorption maxima around 660 nm and well-separated vibronic sidebands at higher photon energies. The steady-state absorption spectra of **PEN**_**2**_, **PEN**_**3**_, and **PEN**_**5**_ are red-shifted compared to the **ssDNA-PEN** spectrum and the ratio between the vibronic sidebands is altered, indicating the expected electronic aggregation of the closely packed pentacenes (Figure 2a). We also observe some spectral broadening which we attribute to the presence of two isomers with the phenylenes in para (lower energy) and meta (higher energy) configurations (see Figure 1a).^35^

In order to track the evolution of excited states, we employed transient absorption (TA) spectroscopy. Here, photoexcitation initially generates a singlet excited state (S_1_) which has a characteristic photoinduced absorption feature at 455 nm (Figure 2b–e).

The spectra of **ssDNA-PEN** do not change their shape with time, indicating that the singlet excited state does not convert into other excited states. This confirms that **ssDNA-PEN** is monomeric when dissolved in a DMSO/buffer mixture.

In the **PEN**_***n***_ constructs, which are composed of electronically coupled pentacenes, the initial photoexcitation rapidly converts to a second species with characteristic absorption at 530 nm (Figure 2c–e). This new absorption can be ascribed to a triplet pair ^1^(T_1_T_1_) species, consistent with previous studies on pentacenes.^8^

Deconvoluting the overlapping spectra using the genetic algorithm yields the species-associated spectra and kinetics given in Supporting Information, Section I. The kinetic fits reveal a more rapid rate of fission with increasing number of coupled pentacenes (Figure 2f). We speculate that this due to singlet delocalization and the increasing probability of the singlet exciton sampling a site optimal for fission along the stack as the number of adjacent pentacenes increases.

Conversion from S_1_ to ^1^(T_1_T_1_) is clear in the TA spectra of the **PEN**_***n***_ samples. However, it is not possible to then track the subsequent fate of the ^1^(T_1_T_1_) state by means of optical spectroscopy, as the spectra overlap with those of possible other species such as quintet ^5^(T_1_T_1_) and separated triplets (T_1_). We employ trESR spectroscopy instead for this purpose, as detailed below.

Nano-/microsecond TA spectroscopy confirms that photoexcited (triplet) species exist in the **PEN**_***n***_ and **(ssDNA-PEN)**_**2**_ samples for several microseconds (Supporting Information, Figure S9). Kinetic decay profiles of the main photoinduced absorption band indicate slightly longer-lived triplets in **PEN**_**3**_ and **(ssDNA-PEN)**_**2**_ (Supporting Information, Figure S10).

**Figure 3:**
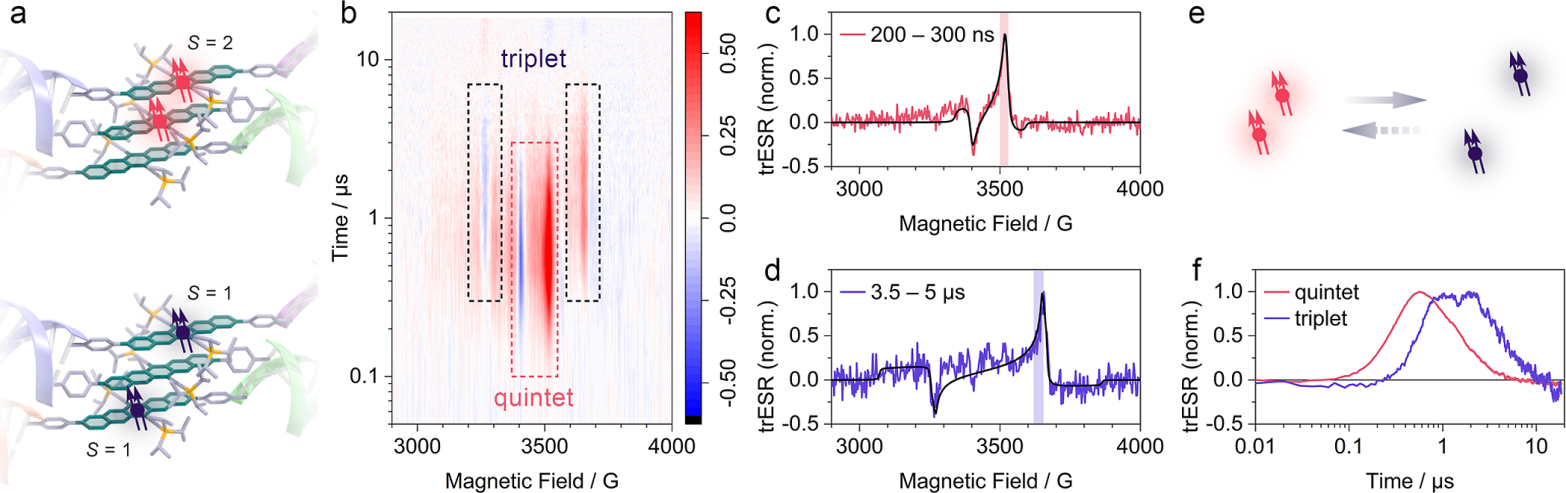
Spin resonance of DNA-assembled TIPS-pentacene trimer. (a) Cartoon representation of possible spin species: the exchange-coupled quintet bi-exciton with spin *S* = 2 (top) and uncoupled triplets each with spin *S* = 1 (bottom). (b) Map of normalized trESR intensity as a function of static magnetic field and time after a 532 nm laser flash. *T* = 50 K. Peaks associated with the quintet (*S* = 2) are highlighted in pink, while peaks associated with the uncoupled triplets (*S* = 1) are marked in black. (c,d) Time-slice integrated from 200–300 ns in (c) and from 3.5–5 μs in (d) each with corresponding fits to quintet and triplet spectra, respectively (see Supporting Information, Section K). (e,f) Pictorial representation and plot of kinetics observed in time-resolved spin polarization of the absorptive triplet peak highlighted in (c) and the quintet peak highlighted in (d).

trESR provides a complementary probe of both coupled and uncoupled triplet excitons and spatially separated or isolated triplets. We focus first on the DNA-assembled trimer, **PEN**_**3**_. As shown in Figure 3a, the trimer structure can house both nearest-neighbor triplet pairs and pairs separated by one pentacene unit to form a next-nearest neighbor pair. At early times, following a 532 nm laser excitation we observe the signatures of exchange-coupled triplet pairs in an overall spin quintet state (marked *S* = 2 in Figure 3b, with corresponding spectral simulation overlayed with experimental spectrum integrated from 200–300 ns as shown in Figure 3c). This early time spectrum is well reproduced by assuming a strongly coupled triplet pair state with total spin *S* = 2, quintet ZFS parameters *D*_q_ = 350 MHz, *E*_q_ < *D*_q_/35, and overpopulation of the *m* = 0 sublevel, where *m* is the spin-projection quantum number along the external magnetic field direction (see Supporting Information, Section K for details of simulation and least-squares fitting). While we discuss the ZFS parameters in more detail later, we note here that the measured quintet spectrum and polarization are consistent with previous observations of quintet formation in covalently linked acene dimers, dilute acene clusters in an insulating matrix, and neat acene films.^12−14,16−18^

This quintet spectrum decays with a time constant of 800 ns (see Supporting Information, Section K) and we observe only uncoupled, free triplets (marked *S* = 1 in Figure 3b, with corresponding spectral simulation overlayed with late-time spectrum in Figure 3d). The ZFS parameters of the free triplet are *D*_t_ = 1.1 GHz and *E*_t_ < *D*_t_/70. As we do not observe any shift in the field position of *S* = 1 features over time, we cannot distinguish between next-nearest neighbor triplet pairs with minimal exchange coupling (within the linewidth of the ESR transitions) and lone triplet excitons formed following the decay of one exciton to the ground state. The observed triplet polarization decays with a lifetime of 1.6 μs (see the Supporting Information). We note that this decay provides a lower bound for the intrinsic triplet polarization lifetime due to contributions from relaxation and microwave driving in the transient experiments performed here. The kinetics shown in Figure 3f are consistent with our interpretation of the trESR spectra, that is, that the outer triplet peaks correspond to uncoupled triplet excitons, the terminal state of fission, while the inner peaks correspond to the initially formed bound triplet pair state following spin mixing from the *S* = 0 coupled pair (see schematic in Figure 3e).

**Figure 4:**
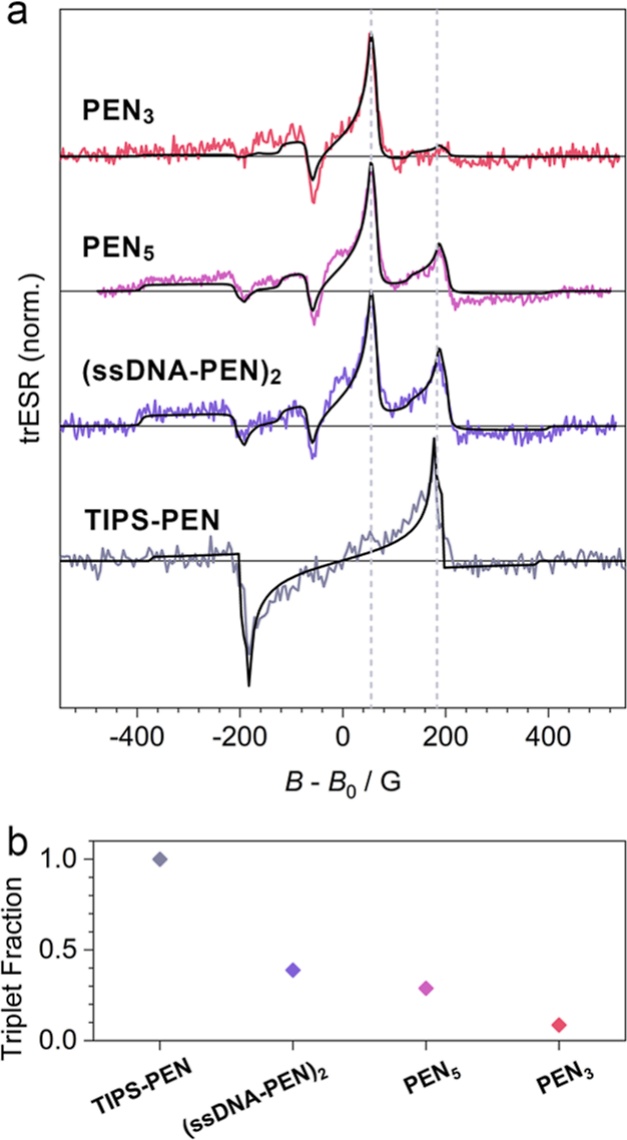
Spin resonance of DNA-assembled and neat TIPS-pentacene. (a) Comparison of the early time spectrum integrated from 250 to 350 ns after laser flash for DNA-assembled pentacene at 50 K and a neat film of **TIPS-PEN** at 10 K. Spectra are offset in field by *B*_0_ = *hf*/*g*μ_b_ where *f* is the applied microwave frequency, *h* the Planck constant, *g* the Landé *g*-factor, and μ_b_ the Bohr magneton. The black line indicates the combined triplet and quintet spectra with relative weights given in (b).

We now compare the observed spin properties of **PEN**_**3**_ to those of **PEN**_**5**_, the self-assembled **(ssDNA-PEN)**_**2**_, and a drop-cast film of 6,13-bis(triisopropylsilylethynyl) pentacene (**TIPS-PEN**). The early time (200–300 ns) trESR spectrum of **TIPS-PEN** also exhibits the expected pattern of SF-borne free triplet excitons with overpopulation of the *m* = 0 state (Figure 4a).^36^ This spectrum can be simulated (black line) with ZFS parameters *D*_t_ = 1.1 GHz and *E*_t_ < *D*_t_/50, consistent with previous reports and with the long-time triplet state observed in the DNA assemblies (see the Supporting Information for details of least-squares spectral fitting).^37−39^ As shown in Figure 4a, the ESR spectra at early times (300 ns after laser flash) for the DNA-assembled structures all show a dominant quintet spectrum and a secondary triplet spectrum. The ZFS parameters of both the triplet and quintet do not vary between DNA-assembled structures (see the Supporting Information for spectral fitting).

In the limit of strong exchange coupling between triplets, the quintet ZFS parameters are determined by the combination of all dipolar interactions between and within the triplet pairs^15^ and thereby provide a link between spin spectrum and pair geometry. This relation allows us to refine the MD-derived structure models of our pentacene stacks as described below.

As detailed in Supporting Information, Section K, we calculate the distribution of quintet ZFS parameters for the range of pentacene pair geometries produced by the MD simulations of the DNA-linked dimer (Supporting Information, Figure S14). Assuming a point-dipole approximation for the triplet at the center of each pentacene core, the quintet ZFS Hamiltonian is given by
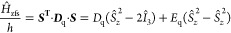
where ***S*** is the vector of quintet spin operators,  is the 3D identity matrix, and  is the spin operator defined along the principal axis  of the ZFS tensor ***D***_q_, determined by the symmetry of the underlying spin–spin interactions. ***D***_q_ can be written in terms of the ZFS parameters of the underlying triplets (on pentacene molecules *a* and *b*) and the dipolar interactions between these triplets.
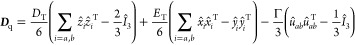
where *D*_T_ and *E*_T_ are the triplet ZFS parameters, , *i* ∈ *a*, *b* are the symmetry axes for the triplets on molecule *a* and *b*.  is the strength of the inter-triplet dipolar interaction where μ_0_ is the magnetic permeability of free space, μ_B_ is the Bohr magneton, *g* is the *g*-factor,  is the center-to-center vector between triplet-bearing molecules, and  is the unit vector between molecules *a* and *b*.

We use the measured triplet ZFS parameters and define  along the long, short, and out-of-plane axes.^40^ The relative orientations of each set of triplet axes and the inter-triplet dipolar interaction strength and unit vector are then taken from MD-calculated geometries (see Supporting Information, Section D for MD simulation details). In Supporting Information, Table S4, we report the resulting theoretical quintet ZFS parameter along with the inter-triplet center-to-center distance. To refine the MD-derived pentacene pair geometry, we determine the range of ZFS parameters that could result from strain in both offset (up to 0.5 Å) and the angle (up to 10°) between each monomer. We find that the observed ZFS parameters could result from slight systematic strain from the MD geometry (see Supporting Information, Section K for further details).

While the observed quintet spectra are consistent with a conserved triplet pair geometry between assemblies, the dynamics of free triplet formation does vary among the different structures. As shown in Figure 4b, the fractional spectral contribution of the triplet to the overall spectrum at 300 ns reduces from **TIPS-PEN** to **PEN**_**3**_, suggesting less efficient initial triplet dissociation as the number of electronically connected monomers is reduced compared to the 3D network formed in neat solid-state films. Unexpectedly, the self-assembled **(ssDNA-PEN)**_**2**_ exhibits more initial triplet polarization intensity than the dsDNA constructs, consistent with the observed increase in the fission rate (see Supporting Information, Section K). This enhanced spin-polarized free triplet formation could be due to the higher degree of rotational and vibrational freedom in ssDNA assemblies (each monomer has a single attachment point to the ssDNA). In all DNA-assembled structures, the free triplet signatures then decay on microsecond timescales with only minor variation in the rate (see Supporting Information, Section K), suggesting a decay pathway intrinsic to the monomer, that is, spin–orbit-mediated decay to the singlet ground state, observed previously in spin resonance measurements of TIPS-tetracene.^41^ These results suggest that the assembly of pentacenes via dsDNA can be used to tune dimensionality of the molecular network to modify free triplet formation while keeping the underlying nearest-neighbor pair-wise interactions conserved due to the strong effect of the side chains on determining the inter-triplet geometry.

## Conclusions

This work presents a method for constructing precisely defined, π-stacked assemblies of SF chromophores using DNA. Using TIPS-modified pentacene as a well-established SF material and equipping it with two ssDNAs of different base sequences has enabled us to control its aggregation to form discrete stacks of exactly defined size.

The pentacene stacks show fast exciton doubling via SF, with the triplet generation rate increasing with the number of coupled pentacenes. Observation of a quintet intermediate state confirms that the SF proceeds via a bound triplet pair state. Analysis of this quintet spectrum provides further insights into the coupling and geometry of this state and allows us to refine the MD-simulated structure models.

Our DNA-encoded assembly of coupled semiconductor molecules allows controlled construction of electronically functional structures. Our findings here of efficient SF through quintet states demonstrate that this design enables efficient and controlled semiconductor operation even in a highly polar environment and point toward future opportunities for constructing functional optoelectronic systems.

## Data Availability

The data underlying this publication are available at https://doi.org/10.17863/CAM.94133.

## Supporting Information Available

Experimental methods, synthetic procedures, and additional spectroscopic data (PDF)
