# Correction to “Photogeneration of Spin Quintet Triplet–Triplet Excitations in DNA-Assembled Pentacene Stacks”

**DOI:** 10.1021/jacs.3c02230

**Published:** 2023-03-31

**Authors:** Sarah R. E. Orsborne, Jeffrey Gorman, Leah R. Weiss, Akshay Sridhar, Naitik A. Panjwani, Giorgio Divitini, Peter Budden, David Palecek, Seán T. J. Ryan, Akshay Rao, Rosana Collepardo-Guevara, Afaf H. El-Sagheer, Tom Brown, Jan Behrends, Richard H. Friend, Florian Auras

Pages 5436. In the analysis and discussion of the ESR experiments, the definition of the quintet zero-field-splitting Hamiltonian was mistakenly typed as
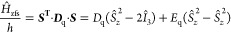


The equation and definition of its terms should read as follows:
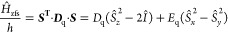
where ***S*** is the vector of quintet spin operators, *Î* is the 5×5 identity matrix, and *Ŝ*_*i*_ is the spin operator defined along the principal axis *i* ∈ (*x̂*,*ŷ*,*ẑ*) of the ZFS tensor ***D***_q_, determined by the symmetry of the underlying spin–spin interactions.

This is also updated in Section K (“Theoretical quintet (*S*=2) zero-field splitting parameters”, page S22) of the Supporting Information.

These corrections do not affect the conclusions presented in the published article.

## Supporting Information Available

Experimental methods, synthetic procedures, and additional spectroscopic data (updated) (PDF)

