# Supplementary material for: Photogeneration of Spin Quintet Triplet–Triplet Excitations in DNA-Assembled Pentacene Stacks
Source: J Am Chem Soc. 2023 Feb 24;145(9):5431–8. doi: 10.1021/jacs.2c13743 (PMC9999418; doi:10.1021/jacs.2c13743)
Supplement: Supplementary file 1 — ja2c13743_si_001.pdf [file ja2c13743_si_001.pdf]

SUPPLEMENTARY INFORMATION

Photogeneration of spin quintet triplet-triplet excitations in DNA-assembled pentacene stacks

Sarah R. E. Orsborne[1], Jeffrey Gorman[1], Leah R. Weiss[2], Akshay Sridhar[3], Naitik A. Panjwani[4], Giorgio Divitini[5], Peter Budden[1], David Palecek[1], Seán T. J. Ryan[1], Akshay Rao[1], Rosana Collepardo-Guevara[1,6], Afaf H. El-Sagheer[7,8,9], Tom Brown[7], Jan Behrends[4], Richard H. Friend[1]*, Florian Auras[1]*

[1] Cavendish Laboratory, Department of Physics, University of Cambridge, CB3 0HE, Cambridge, United Kingdom
[2] Pritzker School of Molecular Engineering, University of Chicago, 60637, Chicago, USA
[3] Department of Applied Physics, Science for Life Laboratory, KTH Royal Institute of Technology, 17121, Solna, Sweden
[4] Berlin Joint EPR Lab, Fachbereich Physik, Freie Universität Berlin, 14195 Berlin, Germany
[5] Department of Materials Science & Metallurgy, University of Cambridge, CB3 0FS, Cambridge, United Kingdom
[6] Yusuf Hamied Department of Chemistry, University of Cambridge, Cambridge, CB2 1EW, United Kingdom
[7] Department of Chemistry, University of Oxford, OX1 3TA, Oxford, United Kingdom
[8] Department of Science and Mathematics, Suez University, Faculty of Petroleum and Mining Eng., Suez 43721, Egypt
[9] current address: School of Chemistry, University, of Southampton, Southampton SO17 1BJ, United Kingdom
*fa355@cam.ac.uk, rhf10@cam.ac.uk

Table of contents

A	Methods . . .	2	H	$(ssDNA\text{-}PEN)_2$ TA spectra . .	17
B	Syntheses . . .	3	I	Genetic algorithm deconvolution .	18
C	DNA design . . .	10	J	Nanosecond TA spectroscopy .	19
D	Molecular dynamics simulations .	11	K	Transient ESR spectroscopy. .	20
E	Self-assembled dimer $(ssDNA\text{-}PEN)_2$	13	L	References . . .	24
F	Modelling of larger constructs .	15			
G	Cryogenic TEM . . .	16			

Abbreviations

DCM dichloromethane

TCA trichloroacetic acid

THF tetrahydrofuran

A. Methods

Nuclear magnetic resonance (NMR) spectra were recorded on Bruker Avance III HD spectrometers. Chemical shifts are expressed in parts per million (δ scale) and are calibrated using residual (undeuterated) solvent signals as an internal reference (^{1}H NMR: $CDCl_3$: 7.26, DMSO-d_6: 2.50; ^{13}C NMR: $CDCl_3$: 77.2, DMSO-d_6: 39.5). Data for ^{1}H NMR spectra are reported in the following way: chemical shift (δ ppm) (multiplicity, coupling constant, integration). Multiplicities are reported as follows: s = singlet, d = doublet, t = triplet, q = quartet, p = quintet, m = multiplet, br = broad, or combinations thereof. ^{13}C NMR assignments were supported by DEPT-135 spectra where necessary.

Infrared (IR) spectra were recorded on Thermo Scientific Nicolet iN10, Perkin-Elmer Spectrum One, and Perkin-Elmer Spectrum BX FT-IR spectrometers equipped with attenuated total reflectance (ATR) units, with internal reference.

High resolution mass spectrometry (HRMS) was carried out using Bruker ultrafleXtreme matrix-assisted laser desorption/ionization time-of-flight (MALDI-TOF) or Waters LCT Premier electrospray ionisation (ESI) mass spectrometers. Reported mass values are within the error limits of ±5 ppm.

Steady-state **UV-vis absorption spectra** were recorded on a Shimadzu UV-3600 spectrometer.

Steady-state photoluminescence (PL) emission spectra were recorded on an Edinburgh Instruments FLS980 spectrometer equipped with a photon counting PMT detector. Absolute **Photoluminescence Quantum Efficiencies (ϕ_F, PLQE)** were determined by using the method from De Mello *et al*.[1] The samples were positioned inside a Spectralon-coated integrating sphere (Newport 819C-SL-5.3) modified with a custom baffle extension. Temperature and current controlled laser diodes (Thorlabs) were used as stable light sources for excitation. Light from the experiment was collected using an optical fibre connected to a Andor Kymera 328i spectrometer housing a DU490A-1.7 InGaAs detector.

For **femtosecond transient absorption spectroscopy (fs-TAS)**, measurements were taken using a Pharos amplifier from Light Conversion (1030 nm, 190 fs, 38 kHz). The pump was the ~250 fs 530 nm output of an ORPHEUS optical parametric amplifier (Light Conversion), while the probe pulses were white light generated by a YAG crystal. Detection was by a custom camera (Entwicklungsbüro Stresing).

For **nanosecond transient absorption (ns-TAS) measurements**, the pump was generated by the output of a Ti:sapphire amplifier system (800 nm, 90 fs, 1 kHz, Solstice Ace), with a Light Conversion TOPAS to generate the desired pump wavelength. The probe was a ~1 ns pulse length supercontinuum laser (LEUKOS Disco STM-1-UV). The probe was split into a probe and reference beam to account for shot-to-shot fluctuations in the probe intensity. The probe and reference beams were focused into an imaging spectrometer (Andor, Shamrock SR 303i) and detected using a pair of linear InGaAs image sensors (Hamamatsu, G11608) driven and read out at the full laser repetition rate by a custom-built board (Entwicklungsbüro Stresing).

Transient electron spin resonance (trESR) spectroscopy. Solutions of the DNA/PEN samples were prepared in a buffer/glycerol solution (80:20 by volume). The solutions were transferred to fused silica ESR tubes, degassed via three freeze-pump-thaw cycles, and backfilled with helium to ca. 500 mbar. The samples were then flame sealed and stored at room temperature in the dark. Prior to the measurements, the samples were flash frozen in liquid nitrogen and placed in a pre-cooled (120 – 150 K) cryostat.

The trESR experiments for **(ssDNA-PEN)$_2$** and **PEN$_3$** were performed on a laboratory-built X-band (9.7 GHz) continuous wave spectrometer together with a critically coupled Bruker MD5 dielectric ring resonator with optical access and implemented direct detection via a detection diode. A 532 nm Nd:YAG pulsed laser (Atum Laser Titan AC compact 15 MM) with a 1 mJ, 5 ns length pulse operating at 100 Hz repetition rate was used to excite the sample in combination with a depolarizer to avoid polarization effects. The temperature was controlled using a Lakeshore 332 temperature controller and helium flow cryostat, samples were measured at 50 K. TIPS-PEN films were measured on the same setup at 10 K.

The trESR experiments for **PEN$_5$** were performed at X-band (9.7GHz) on a Bruker ElexSys E580 spectrometer together with a critically coupled MD5 dielectric ring resonator with optical access and implemented direct detection via an IQ mixer. The sample was excited at 605 nm using a Opta OPO (Model 355 I, 410-700 nm) pumped by the 355 nm output of a Spectra-Physics QuantaRay LabSeries 150 Nd:YAG laser. The incident 605 nm laser pulse energy was 7.5 mJ with a 7 ns length pulse operating at 10 Hz repetition rate. A depolarizer was used to avoid polarization effects. The temperature was controlled using a helium flow cryostat and an ITC temperature controller from Oxford Instruments.

Transients were recorded as the static magnetic field was swept and continuous-wave microwave irradiation was applied. All DNA/PEN assemblies were measured with a microwave power of 0.63 mW. We expect the time resolution of both setups to be limited by the Q-factor of the critically coupled MD5 resonator and therefore do not expect any significant effect on the kinetics due to the detection method (diode vs IQ mixer).
For analysis, EPR spectra were simulated using EasySpin.[2]

Cryo-TEM. Using a standard vitrification procedure, samples were plunge-frozen into liquid ethane in a ThermoFisher Vitrobot Mk3 on Quantifoil TEM grids. The TEM imaging was carried out in low electron dose conditions in a ThermoFisher Krios G3i operated at 300 kV using a Falcon3 camera, controlling acquisition using EPU.

DNA synthesis

DNA synthesis was performed on an Applied Biosystems 394 automated DNA/RNA synthesizer using a standard phosphoramidite cycle of detritylation, coupling, capping and oxidation using TCA (3% in DCM), 1*H*-tetrazole (0.45 M in MeCN), Cap A (10% acetic anhydride, 10% lutidine and 80% THF) / Cap B (16% N-methylimidazole in THF) and iodine (0.02 M in THF, pyridine and H_2O) on a 1.0 µmol scale. β-cyanoethyl protected phosphoramidites (dA-bz, dG-ib, dC-bz and dT where bz = benzoyl and ib = iso-butyryl, Sigma-Aldrich) were dissolved in anhydrous MeCN (0.1 M) immediately prior to use. The coupling time for dA, dC, dG and dT monomers was 45 s, and 10 min for 5-octadiynyl-dU CE-phosphoramidite (BA0308-F100 from Biosearch Technologies, dissolved in anhydrous MeCN (0.1 M) immediately prior to use). Stepwise coupling efficiencies were determined by automated trityl cation conductivity monitoring and were >98% in all cases. 3'-Octadiynyl oligonucleotides were made with 3'-phosphate or 3"-OH.

Cleavage of the oligonucleotides from the solid support and subsequent deprotection was achieved by exposure to concentrated aqueous ammonia solution for 1 h at room temperature, followed by heating in a sealed tube for 5 h at 55 °C.

The oligonucleotides were purified by reversed-phase HPLC on a Gilson system using a Luna 10 µm ACE® C8 100 Å pore Phenomenex column (10 × 250 mm) with a gradient of MeCN in ammonium acetate or triethylammonium bicarbonate (TEAB) over 20 min at a flow rate of 4 mL min^{-1}. TEAB buffers (buffer A: 0.1 M TEAB, pH 7.5; buffer B: 0.1 M TEAB, pH 7.5, with 50% MeCN) were used (0% to 80% buffer B over 20 min). Elution was monitored by UV absorption at 295 nm.

All oligonucleotides were characterised by negative-mode electrospray using a UPLC-MS Waters XEVO G2-QTOF mass spectrometer and an Acquity UPLC system with a BEH C18 1.7 µm column (Waters). A gradient of triethylamine (TEA) and hexafluoroisopropanol (HFIP) in MeOH was used (buffer A, 8.6 mM TEA, 200 mM HFIP in 5% MeOH/H_2O (v/v); buffer B, 20% v/v buffer A in MeOH). Buffer B was increased from 0–70% over 7.5 min or 15–30% over 12.5 min for normal oligonucleotides and 50–100% over 7.5 min for hydrophobic oligonucleotides. The flow rate was set to 0.2 mL/min. Raw data were processed and deconvoluted using the deconvolution software MassLynx v4.1.

B. Syntheses

Reagents and solvents were obtained in high-purity grades from commercial suppliers. DNA strands were purchased from IDT and diluted in PBS (20 mM phosphate buffer, 200 mM NaCl). Alkyne-terminating, 12-mer ssDNA, with all protecting groups on and on-resin, was purchased from IDT and used as received. Chemical syntheses were carried out in oven-dried glassware under an argon atmosphere unless otherwise stated. Reactions were followed by analytical thin layer chromatography on aluminium-backed silica gel plates (Merck, 60 Å, F_{254}).) and visualised with ultraviolet irradiation (λ_{max} = 254 or 365 nm) or permanganate staining. Flash chromatography purification was carried out with Acros Organics ultra-pure silica gel (60 Å, 40 – 60 µm) under a positive pressure of air.

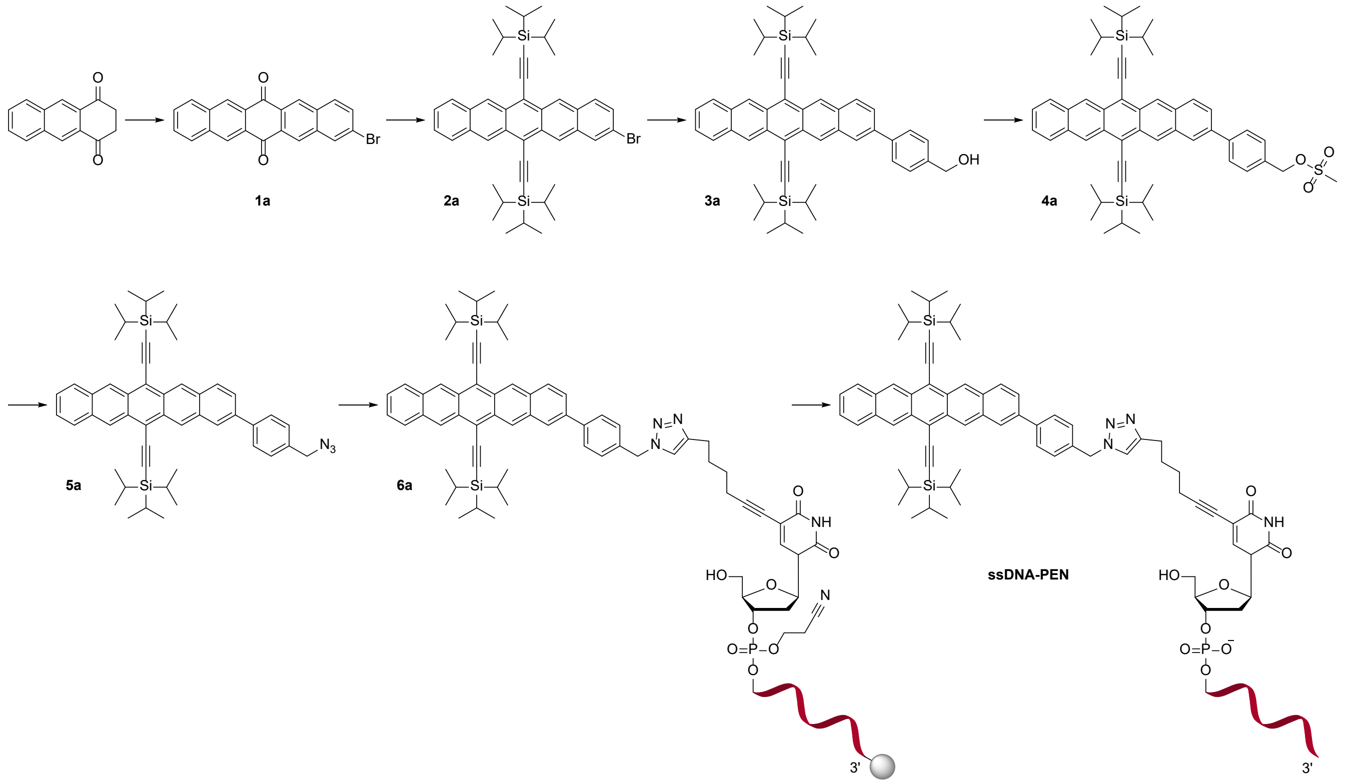

Scheme 1. Synthesis of the **ssDNA-PEN** monomer.

Compound 1a

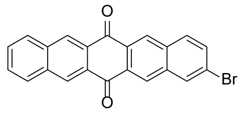

In accordance with literature.[3] 4-Bromo-1,2-bis(dibromomethyl)benzene (1 g, 4.81 mmol, 1 eq.), 1,4-anthraquinone (2.5 g, 5 mmol, 0.9 eq.), and KI (3.27 g, 20 mmol, 5 eq.) were mixed in DMF (40 mL) at room temperature and heated to 110 °C under argon overnight. The suspension was cooled to 0 °C, filtered, and washed sequentially with MeOH, H_2O, and MeOH + 5% $CHCl_3$ to yield **1a** as a gold-brown insoluble powder (crude yield: 1.03 g, 2.66 mmol, 55 %).

No chemical characterisation was performed due to the insolubility in common organic solvents.

Compound 2a

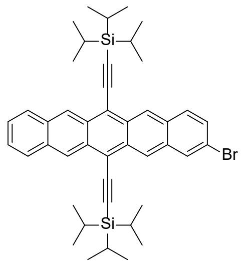

In accordance with literature.[3] Quinone **1a** (583 mg, 1.51 mmol, 1 eq.) was dispersed in THF (20 mL) at -78 °C. In a separate flask, 2.5 M *n*-BuLi in hexanes (2.16 mL, 5.4 mmol, 3.6 eq.) was added dropwise to a stirred solution of (triisopropylsilyl)acetylene (1.17 mL, 5.26 mmol, 3.5 eq.) in THF (30 mL) at -78 °C under argon and stirred for 1 h. The lithiated mixture was transferred to a dispersion of **1a** by cannula at -78 °C. The reaction was allowed to warm to room temperature and stirred overnight. 5 mL of saturated $SnCl_2{\cdot}2H_2O$ in degassed 10% HCl was added by injection to the crude. The crude mixture was degassed for a further 5 min, then left to stir for 3 h. The reaction mixture was filtered under reduced pressure through Celite® with DCM. The eluent was washed with water,

then brine, dried over $MgSO_4$, filtered, and concentrated under reduced pressure. The solid was purified by column chromatography (silica gel, heptane) to yield **2a** as a blue solid (832 mg, 1.16 mmol, 77%).

^{1}H NMR (400 MHz, $CDCl_3$): δ 9.30 (d, J = 5.7 Hz, 2H), 9.26 (s, 1H), 9.19 (s, 1H), 8.13 (s, 1H), 7.97 (m, 2H), 7.86 – 7.80 (m, 1H), 7.49 – 7.40 (m, 3H), 1.40 – 1.35 (m, 38H).

^{13}C NMR (101 MHz, $CDCl_3$): δ 132.6, 132.5, 132.2, 130.9, 130.8, 130.6, 130.5, 130.4, 130.2, 130.1, 129.5, 128.7, 126.9, 126.4, 126.3, 126.2, 126.1, 120.3, 118.7, 118.5, 107.6, 107.5, 104.4, 104.3, 19.0, 18.9, 11.6.

R_f = 0.41 (heptane).

Compound 3a

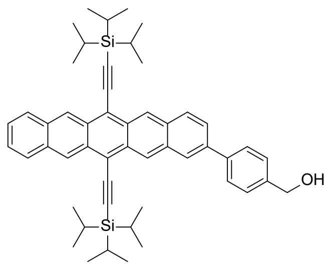

Pentacene **2a** (720 mg, 1 mmol, 1 eq.), SPhos (11 mg, 0.025 mmol, 2.5 mol%), K_2CO_3 (276 mg, 2 mmol, 2 eq.), $Pd_2(dba)_3{\cdot}CHCl_3$ (14 mg, 0.013 mmol, 1.3 mol%), and 4-(hydroxymethyl)phenylboronic acid pinacol ester (360 mg, 1.5 mmol, 1.5 eq.) were mixed in 5 mL of degassed toluene/H_2O (4:1, *v*:*v*), and stirred under argon at 100 °C for 4 h. The reaction was diluted with water and extracted into DCM. The combined organic phases were washed with brine, dried over $MgSO_4$, filtered, and concentrated under reduced pressure. The solid was purified by column chromatography (silica gel, DCM) to yield **3a** as a blue solid (564 mg, 0.76 mmol, 76%).

^{1}H NMR (400 MHz, $CDCl_3$): δ 9.36 – 9.29 (m, 4H), 8.13 (s, 1H), 8.08 – 8.03 (m, 1H), 8.01 – 7.94 (m, 2H), 7.83 – 7.79 (m, 2H), 7.73 – 7.69 (m, 1H), 7.57 – 7.52 (m, 2H), 7.45 – 7.39 (m, 2H), 4.81 (d, J = 5.9 Hz, 2H), 1.50 – 1.11 (m, 42H).

^{13}C NMR (101 MHz, $CDCl_3$): δ 140.3, 140.2, 137.9, 132.4, 132.4, 132.3, 131.5, 131.0, 130.8, 130.7, 130.6, 129.4, 129.0, 128.7, 128.4, 127.6, 127.5, 126.6, 126.3, 126.2, 126.1, 118.5, 118.3, 101.7, 101.7, 104.7, 104.6, 65.2, 19.0, 19.0, 11.7.

R_f = 0.39 (DCM). HRMS (ESI) *m*/*z*: $[M + H]^+$ calc for $C_{51}H_{61}OSi_2^+$ 744.4209; found 744.4177.

Compound 4a

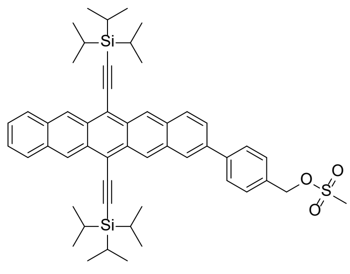

Pentacene **3a** (300 mg, 0.41 mmol, 1 eq.) and Et_3N (86 µL, 0.615 mmol, 1.5 eq.) were mixed in 40 mL DCM under argon at 0 °C. Methanesulfonyl chloride (82 µL, 0.48 mmol, 1.1 eq.) was added by injection; the reaction was allowed to warm to room temperature and stirred for 1.5 h. The mixture was diluted with H_2O (400 mL) and extracted with DCM. The combined organic phases were washed with brine, dried over $MgSO_4$, filtered, and dried under reduced pressure. The blue solid was used immediately in the next step without further purification.

R_f = 0.65 (DCM)

Compound 5a

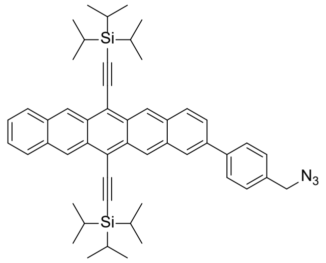

Pentacene **4a** (ca. 0.40 mmol, 1 eq.) was dissolved in DMF (30 mL) under argon at room temperature NaN_3 (31.2 mg, 0.48 mmol, 1.1 eq.) was added as a solid in one portion and stirred overnight. The mixture was diluted with H_2O (400 mL) and extracted with $CHCl_3$. The combined organic phases were washed with brine, dried over $MgSO_4$, filtered, and concentrated under reduced pressure. The solid was purified by column chromatography (silica gel, DCM/heptane 9:1) to yield **5a** as a blue solid (174 mg, 0.23 mmol, 55 % over two steps).

^{1}H NMR (400 MHz, $CDCl_3$): δ 9.34 (s, 1H), 9.32 – 9.30 (m, 3H), 8.14 (s, 1H), 8.06 (d, J = 9.1 Hz, 1H), 8.00 – 7.96 (m, 2H), 7.83 – 7.81 (m, 2H), 7.70 (dd, J = 9.1 Hz, J = 1.8 Hz, 1H), 7.51 – 7.48 (m 2H), 7.42 (m, 2H), 4.45 (s, 2H), 1.45 – 1.37 (m, 42H).

^{13}C NMR (126 MHz, $CDCl_3$): δ 140.9, 137.6, 134.8, 132.3, 132.2, 131.4, 130.9, 130.8, 130.7, 130.6, 129.5, 128.9, 128.7, 127.7, 126.7, 126.7, 126.6, 126.3, 126.3, 126.2, 126.2, 126.1, 126.1, 126.1, 126.0, 126.0, 118.5, 118.3, 107.3, 107.2, 104.6, 104.5, 54.6, 19.0, 19.0, 11.7.

R_f = 0.75 (DCM/heptane 95:5). HRMS (ESI) *m/z*: $[M]^-$ calc for $C_{51}H_{59}N_3Si_2^-$ 770.4320; found 770.4312.

Compound 6a

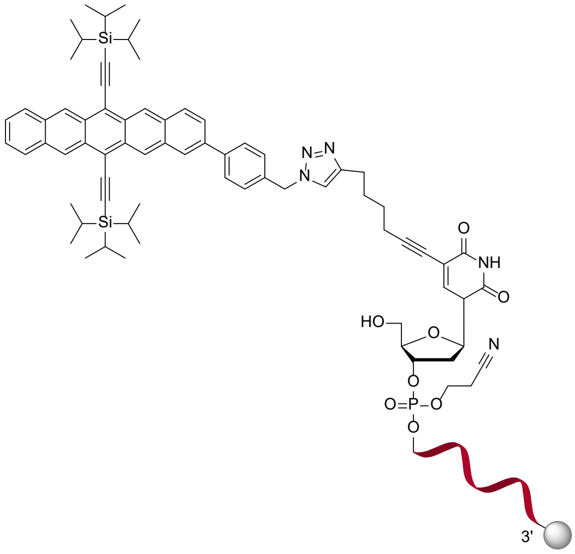

Resin-bound alkyne-terminating 12-mer ssDNA (15 nmol/mg, 2.1 mg, 31.5 nmol, 1 eq.) and **5a** (2.5 mg, 3.15 µmol, 50 eq.) were mixed as solids in an argon-filled 300 µL Eppendorf® tube. The solids were degassed under gentle argon flow. In a separate flask, a 0.1 M tris(benzyltriazolylmethyl)amine solution in DCM was degassed and 0.5 µL (1.5 eq.) was added to the ssDNA-resin/**5a** mixture. Simultaneously, in another flask, 0.1 M CuBr in MeCN solution was degassed, and 0.34 µL (1.1 eq.) was added to the ssDNA-resin/**5a** mixture. The Eppendorf® tube was degassed under argon flow, sealed with PTFE tape and Parafilm®, and shaken for 2 d at room temperature. The crude was spun down to form a resin pellet. The supernatant containing residual **5a** was extracted and separately purified by Chelex® to recover pure **5a**. The blue resin was washed with DCM and the supernatant removed until the washings ran colourless. The blue resin was washed with MeCN three times. Finally, the blue resin was washed twice with ether and left to dry, yielding **6a** as a blue-stained resin.

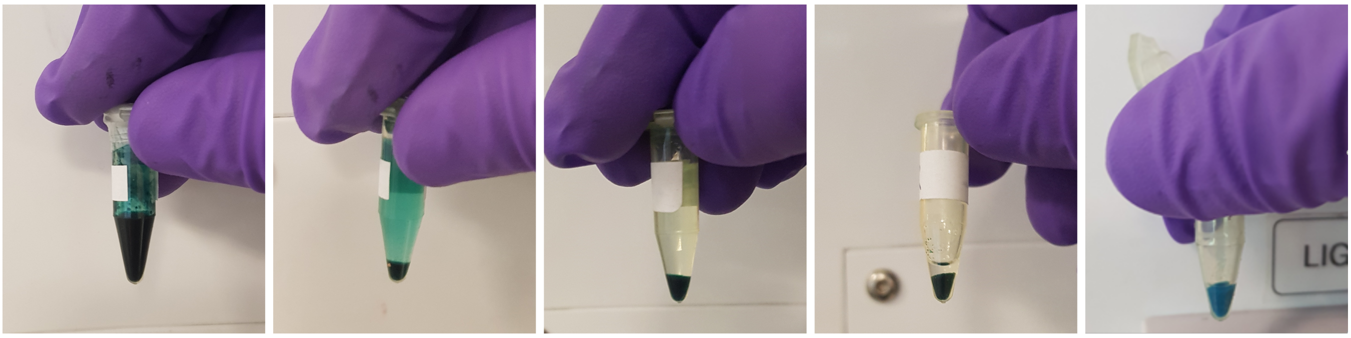

Illustration of the purification/washing steps of the resin-bound pentacene **6a**.

ssDNA-PEN

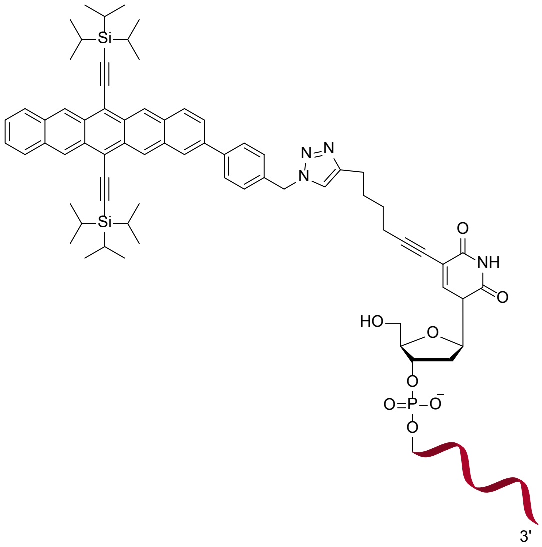

1 mL of a degassed and argon-saturated ammonium hydroxide solution was added to **6a** in a 300 µL Eppendorf® tube to form a dispersion. The dispersion was degassed, and the crude left to shake at room temperature overnight. The blue crude solution was spun down by mini-centrifuge. The ammonium solution was diluted by half with NaCl (100 mg/mL). The blue mixture was loaded onto a Glen-Pack™ DNA purification cartridge (pre-washed with MeCN and TEAA). The column-loaded crude mixture was washed with brine, then detritylated with 2% TFA. Trityl was washed away with ultrapure H_2O, and finally the blue conjugate **ssDNA-PEN** was eluted in MeCN/H_2O (1:1). The resulting blue solutions were lyophilized. Finally, the blue solids were redissolved in PBS to yield **ssDNA-PEN** as an aqueous, blue solution.

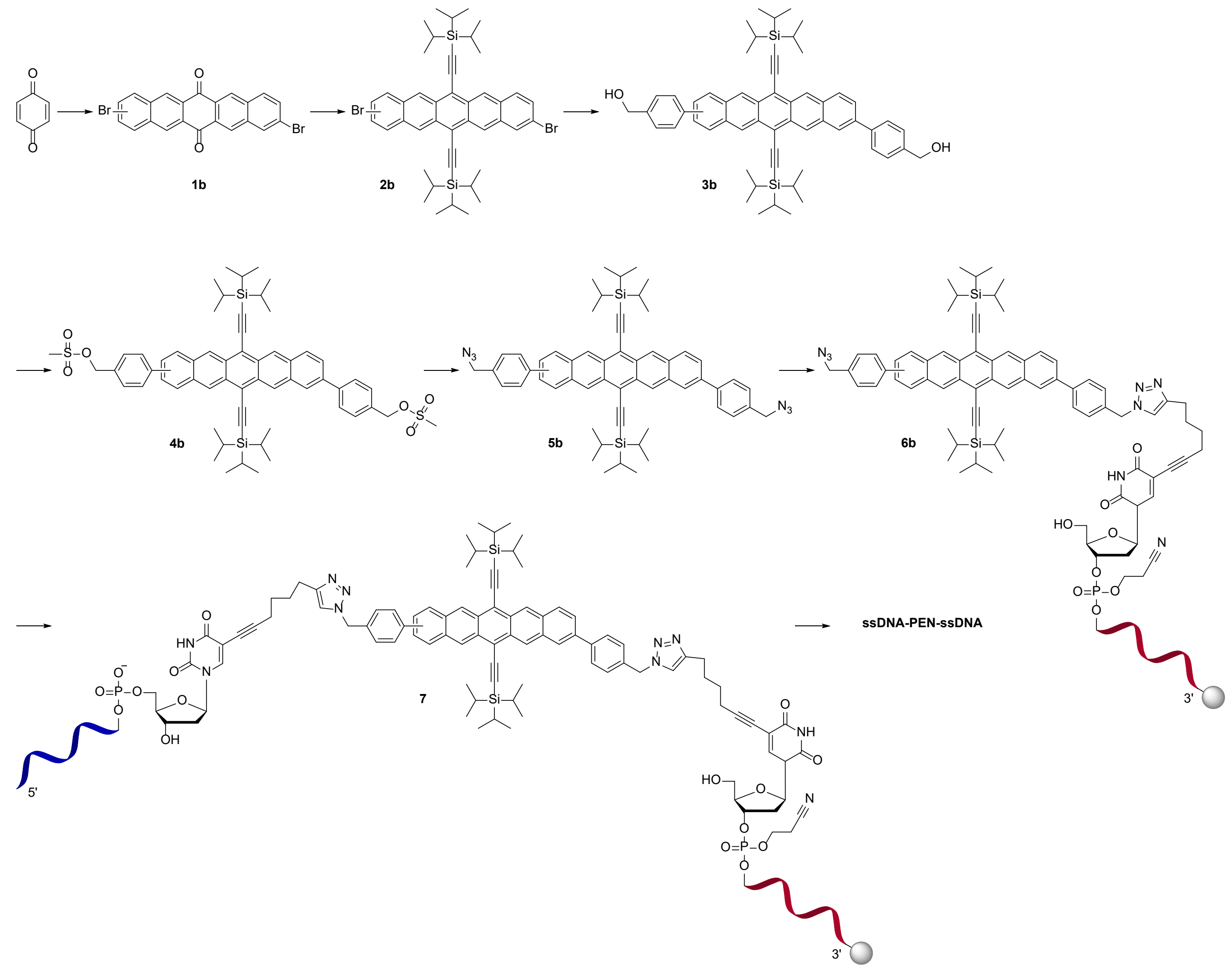

Scheme 2. Synthesis of the **ssDNA-PEN-ssDNA** strands.

Compounds **1b** and **2b** were synthesised as reported previously.[4]

Compound 3b

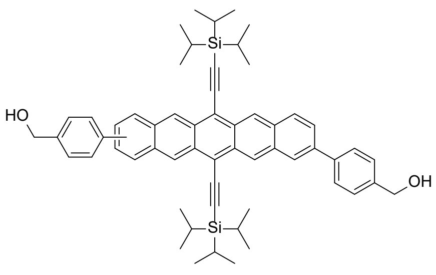

Pentacene **2b** (500 mg, 0.62 mmol, 1 eq.), SPhos (21 mg, 0.05 mmol, 8 mol%), K_2CO_3 (193 mg, 1.4 mmol, 2.3 eq.), $Pd_2(dba)_3 \cdot CHCl_3$ (26 mg, 0.025 mmol, 4 mol%), and 4-(hydroxymethyl)phenylboronic acid pinacol ester (283 mg, 1.86 mmol, 3 eq.) were mixed in 5 mL of degassed *o*-xylene/H_2O (4:1, *v*:*v*) and stirred under argon at 100 °C for 4 h. The reaction was diluted with water, extracted into DCM. The combined organic phases were washed with brine, dried over $MgSO_4$, filtered, and concentrated under reduced pressure. The solid was purified by column chromatography (silica gel, gradient heptane to DCM/EtOAc 4:1) to yield **3b** as a blue solid (303 mg, 0.35 mmol, 56%).

^{1}H NMR (400 MHz, $CDCl_3$): δ 9.32 – 3.31 (m, 3H), 8.02 – 7.96 (m, 4H), 7.56 – 7.35 (m, 4H), 7.30 (s, 7H), 4.73 (d, *J* = 5.5 Hz, 4H), 2.96 (s, 1H), 2.88 (s, 1H), 1.40 – 1.34 (m, 32 H), 1.25 – 1.24 (m, 10H).

^{13}C NMR (101 MHz, $CDCl_3$): δ 140.6, 139.4, 138.1, 134.9, 132.4, 130.0, 128.6, 126.7, 125.6, 126.2, 126.1, 123.7, 118.4, 65.0, 19.1, 117.2.

R_f = 0.2 (DCM/EtOAc 4:1).

HRMS (ESI) *m*/*z*: $[M + H]^+$ calc for $C_{58}H_{67}O_2Si_2^+$ 851.4675; found 851.4715.

Compound 4b

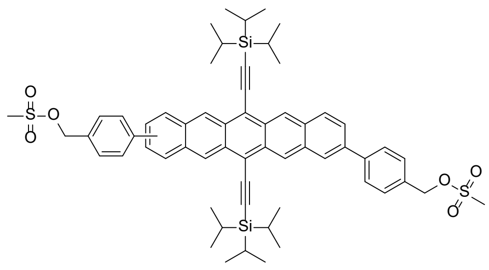

Pentacene **3b** (81 mg, 0.1 mmol, 1 eq.) and Et_3N (18 µL, 0.23 mmol, 2.3 eq.) were mixed in 40 mL DCM under argon at 0 °C. Methanesulfonyl chloride (28 µL, 0.2 mmol, 2 eq.) was added by injection; the reaction was allowed to warm to room temperature and stirred for 1.5 h. The mixture was diluted with H_2O (400 mL) and extracted with DCM. The combined organic phases were washed with brine, dried over $MgSO_4$, filtered, and dried under reduced pressure. The blue solid was used immediately in the next step without further purification.

R_f = 0.9 (DCM/EtOAc 4:1)

Compound 5b

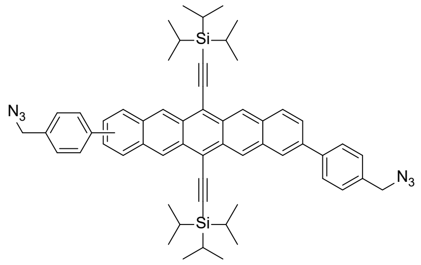

Compound **4b** (ca. 0.1 mmol, 1eq.) was dissolved in DMF (8 mL) under argon at room temperature.NaN_3 (15 mg, 0.23 mmol, 2.3 eq.) was added as a solid in one portion and stirred overnight. The mixture was diluted with H_2O (400 mL) and extracted with $CHCl_3$. The combined organic phases were washed with brine, dried over $MgSO_4$, filtered, and concentrated under reduced pressure. The solid was purified by column chromatography (silica gel, gradient DCM/EtOAc/MeOH 80:20:0 to 100:0:3) to yield **5b** as a blue solid (41 mg, 0.045 mmol, 45% over two steps).

^{1}H NMR (400 MHz, $CDCl_3$): δ 9.34 (d, J = 4.8 Hz, 4H), 8.01 – 7.97 (m, 4H), 7.45 – 7.41 (m, 2H), 7.27 (q, J = 8.2Hz, 8H), 4.36 (s, 4H), 1.43 – 1.35 (m, 42H).

^{13}C NMR (101 MHz, $CDCl_3$): δ 141.2, 139.0, 133.9, 132.4, 131.6, 131.0, 130.8, 130.3, 129.9, 128.7, 128.0, 126.4, 126.3, 126.1, 118.5, 107.4, 104.6, 54.5, 19.0, 11.7.

Rf = 0.65 (DCM/heptane 1:1).

HRMS (ESI) m/z: $[M + H]^+$ calc for $C_{58}H_{65}N_6Si_2^+$ 901.4809; found 901.4760.

Compound 6b

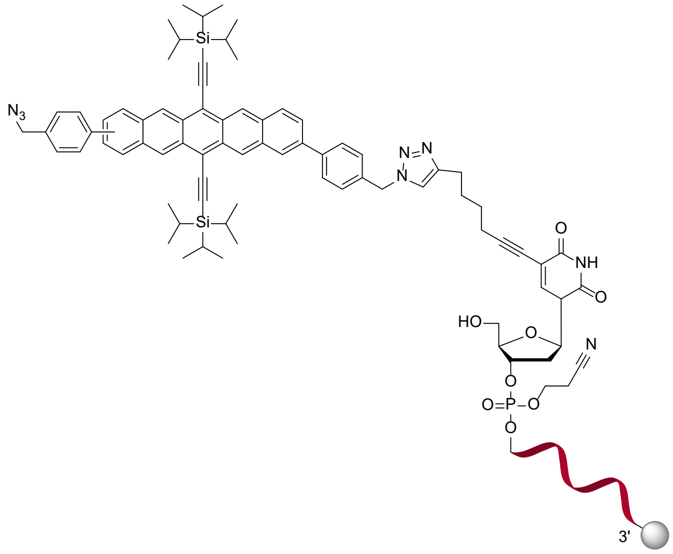

Compound **6b** was prepared by mixing azide **5b** and resin-bound alkyne-terminating 12-mer ssDNA in a 300 µL Eppendorf® tube, and following the synthesis procedure and purification method described above for compound **6a**.

Compound 7

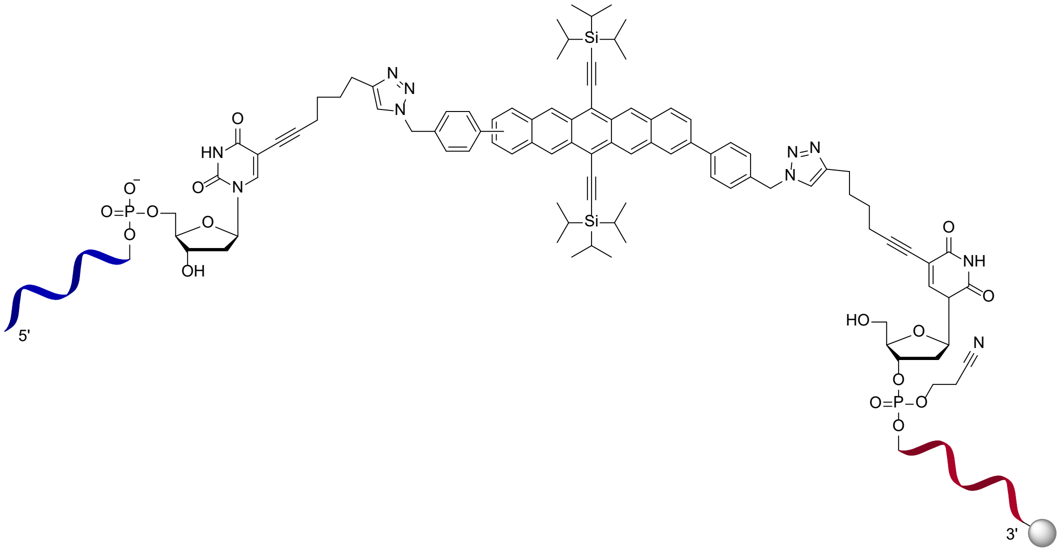

In three separate 300 µL Eppendorf® tubes: 1 M $CuSO_4 \cdot 5H_2O$ in ultrapure H_2O, 1 M tris(3-hydroxypropyltriazolylmethyl)amine in ultrapure H_2O, and 2 M Na-ascorbate in ultrapure H_2O. In another 300 µL Eppendorf® tubes **6b** was kept under argon, and 3'-alkyne DNA (2 eq) in ultrapure H_2O was added, and the suspension degassed for 5 mins. The solutions of $CuSO_4 \cdot 5H_2O$, tris(3-hydroxypropyltriazolylmethyl)amine and Na-ascorbate were mixed in a 1:2:4 volume ratio and degassed, turning from blue to yellow. 10 eq. of this mixture were added to **6b** and the suspension degassed for 5 mins. The Eppendorf® tube was degassed under argon flow, sealed with PTFE tape and Parafilm®, and shaken for 2 d at room temperature The crude was spun down to form a resin pellet. The supernatant was removed. The blue resin was washed with ultrapure H_2O, shaken, spun down and the supernatant removed. This was repeated three times with ultrapure H_2O, and three times with MeCN. Finally, the blue resin was washed twice with ether and left to dry, yielding compound **7** as a blue-stained resin.

ssDNA-PEN-ssDNA

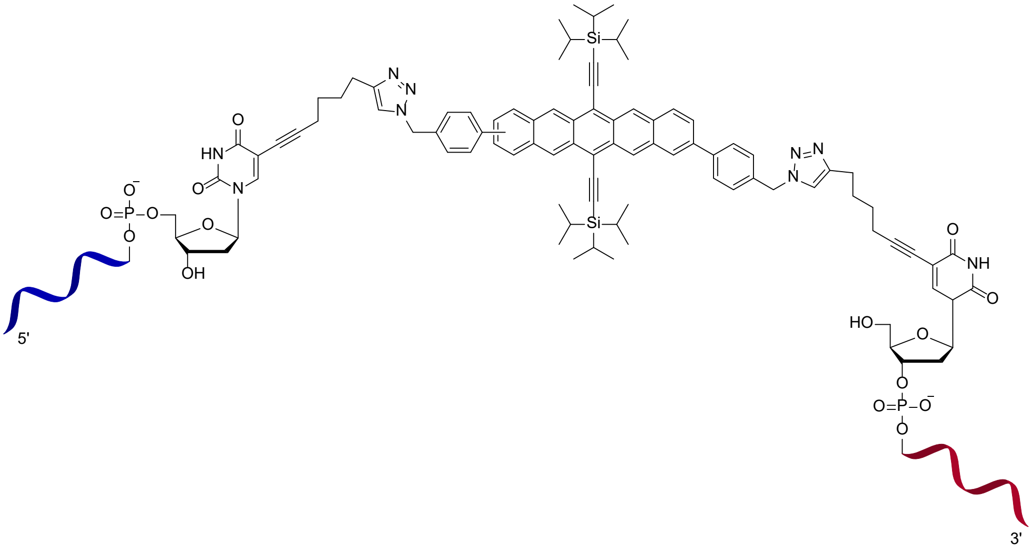

ssDNA-PEN-ssDNA was synthesised and purified as described above for **ssDNA-PEN**.

C. DNA design

The ssDNA-based pentacene samples discussed in this study are constructed with a 12 nucleotide (nt) ssDNA linked via a phenylene and triazole bridge to the 2 position of the pentacene core (see SI, Section B for the synthesis and chemical structures).

The dsDNA-linked constructs in this study are formed from up to seven partially complementary ssDNA strands. Each position within the constructs corresponds to two specific 12 nt sequences (11 nt for terminal strands) on either side of the pentacene cargo – see base sequences below. Pairwise complementary DNA strands are colour coded.

Table S1. Design of the dsDNA-linked pentamer **PEN$_5$**.

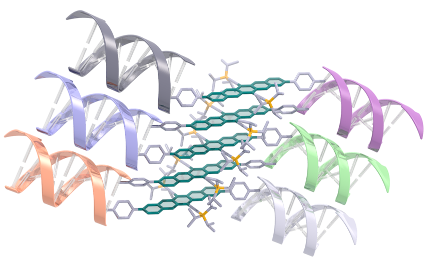

Strand	Sequence
1_{cap2}	3′ CTG GCT CAA TA 5′
2_{PEN}	5′ GAC CGA GTT ATU≡PEN≡UTA ACC AGT GGA 3′
3_{PEN}	3′ CAA GGC GGA TAU≡PEN≡UAT TGG TCA CCT 5′
4_{PEN}	5′ GTT CCG CCT ATU≡PEN≡UAT TCT GTC TGG 3′
5_{PEN}	3′ GGA CAT CCT TTU≡PEN≡UTA AGA CAG ACC 5′
6_{PEN}	5′ CCT GTA GGA AAU≡PEN≡UTT AGG TGC AAG 3′
7_{cap1}	3′ AA TCC ACG TTC 5′

Table S2. Design of the dsDNA-linked trimer **PEN$_3$**.

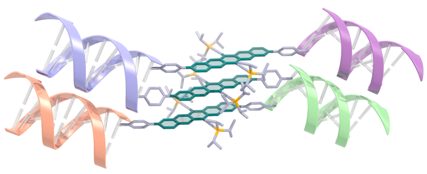

Strand	Sequence
2_{cap2}	5′ TA ACC AGT GGA 3′
3_{PEN}	3′ CAA GGC GGA TAU≡PEN≡UAT TGG TCA CCT 5′
4_{PEN}	5′ GTT CCG CCT ATU≡PEN≡UAT TCT GTC TGG 3′
5_{PEN}	3′ GGA CAT CCT TTU≡PEN≡UTA AGA CAG ACC 5′
6_{cap1}	5′ CCT GTA GGA AA 3′

Table S3. Design of the dsDNA-linked dimer **PEN$_2$**.

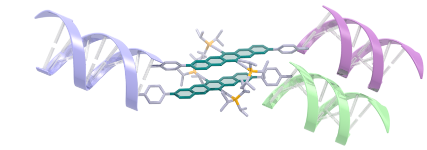

Strand	Sequence
2_{cap2}	5′ TA ACC AGT GGA 3′
3_{PEN}	3′ CAA GGC GGA TAU≡PEN≡UAT TGG TCA CCT 5′
4_{PEN}	5′ GTT CCG CCT ATU≡PEN≡UAT TCT GTC TGG 3′
5_{cap1}	3′ TA AGA CAG ACC 5′

Table S4. Base sequence of the monomeric **ssDNA-PEN**.

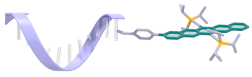

Strand	Sequence
3^{*}_{PEN}	3′ CAA GGC GGA TAU≡PEN 5′

Table S5. Base sequence of the self-assembled dimer **(ssDNA-PEN)$_2$**. This dimer is composed of two identical PEN-modified ssDNA which dimerise due to amphiphilic contrast in aqueous buffer solution.

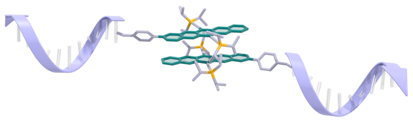

Strand	Sequence
3^{*}_{PEN}	3′ CAA GGC GGA TAU≡PEN 5′
3^{*}_{PEN}	5′ PEN≡UAT AGG CGG AAC 3′

D. Atomistic metadynamics molecular dynamics simulations

Parameterisation

The parameters for pentacene cargo bound to one/two strands of DNA were developed separately. In each case, the structures were sketched using Marvin and hydrogen atoms were added using Chimera.[5] The PyRed server[6] was used to calculate the electrostatic potential of the structures at the HF/6-31G* level of theory using Gaussian09, and then perform a two-stage Restrained Electrostatic Potential (RESP) fit[7] to calculate the atomic charges. Antechamber[8] was then used to assign atom types to the semiconductor/linkers according to the GAFF2 parameter set.[9] Bonded and non-bonded interactions of the Silyl groups were modelled using the parameters of Dong *et al*.[10]

ds-DNA and ss-DNA strands were built 6 basepairs long using Avogadro.[11] The xleap module of Amber16[12] was then used to append pentacene to the DNA strands. The DNA strands were parameterized using the parmbsc1[13] parameters. The system was solvated in an octahedral box of TIP3P[14] solvent and 0.15 M NaCl ions that used the parameters of Joung and Cheatham.[15] Finally, the amber parameters were converted to Gromacs[16] format using Parmed.[17]

Simulation setup

Models were first energy minimized until the maximum force on any atom was below 1000 kJ mol^{-1} nm^{-1}. The systems were then equilibrated using restraints on the DNA and semiconductors (force constants of 1000 kJ mol^{-1} nm^{-2}) for 1 ns in the NVT ensemble followed by 1 ns in the NPT ensemble.

The simulations were performed starting with random velocities obtained from a Maxwell–Boltzmann distribution at 300 K and using a pressure of 1 bar. The temperature was kept constant using the V-rescale thermostat.[18] The pressure was maintained using the Parrinello-Rahman barostat.[19] Long-range electrostatics were calculated using the particle mesh Ewald (PME) algorithm[20-21] with a cutoff of 1.0 nm. The simulations used a timestep of 2 fs and were performed using Gromacs 2021[16] patched with Plumed 2.7.2.[22-23] Trajectories were analysed using a combination of Gromacs and Plumed tools together with Python MDAnalysis[24] scripts.

Metadynamics simulations

Following equilibration, well-tempered metadynamics simulations[25-26] were performed to investigate the relative interactions of Pentacene molecules. Metadynamics simulations promote sampling along collective variable (ξ) space through the addition of an external biasing potential V_E constructed as a sum of gaussians as

$$V_E = \sum_{\{t=\tau_G, 2\tau_G, \dots\}} W \prod_i e^{\left(-\frac{(\xi_i - \xi_{ti})}{2\delta_i^2}\right)^2}$$

where τ_G is the time interval at which the gaussians are added with height W, width δ and mean ξ_{ti}. Here, we used two collective variables (i = 2) that determine the relative stacking orientations of the semiconductors and shown by our prior work[27] to promote sufficient sampling. ξ_1 (also denoted as r) is defined as the Euclidean distance between the centres of the central aromatic rings of the two pentacene molecules. ξ_2 (also denoted as θ) is defined as the angle between the longitudinal axes of the two pentacene molecules (SI, Figure S1a).

The metadynamics biasing potentials were deposited every 10 ps using a bias factor of 15 and gaussian widths of 0.002 nm and 0.005 radians along ξ_1 and ξ_2 respectively. The simulations were performed for 100 ns and convergence was assessed using the time-evolution of the free-energy profiles, which are invariant after ~50 ns (disregarding the time-dependant constant) (SI, Figure S1b).

Normalized probability density calculations

The separation between pentacene molecules were calculated by projecting the distance between the centres of the central aromatic rings along long x and short y molecular axes (SI, Figure S1c). However, the addition of the history dependent bias potentials in metadynamics simulations precludes a direct ensemble averaging of the system's characteristics as simulation time is without physical meaning. Here, we used the methodology of Bonomi *et al*.[28] to

reweight trajectory frames and calculate unbiased equilibrium ensembles. Briefly, the probability distribution of a biased system along molecular coordinates ***r*** can be expressed as

$$P(\vec{r}) = \frac{exp(-\beta[U(\vec{r}) + V_E])}{\int exp(-\beta[U(\vec{r}) + V_E])dr}$$

where U(***r***) is the underlying force field potential and β is the thermodynamic temperature. Disregarding the time-dependent bias offset and assuming convergence along the collective-variable space, this biased probability density can be related to the unbiased probability density P_0(***r***) as

$$P(\vec{r}) = P_0(\vec{r}) \cdot exp(-\beta[V_E(\xi(\vec{r}))])$$

Thus, the offset projections between the two pentacenes (Δ_x and Δ_y) were first calculated for each trajectory frame and subsequently used to calculate a two-dimensional weighted histogram to obtain the normalized probability density (NPD).

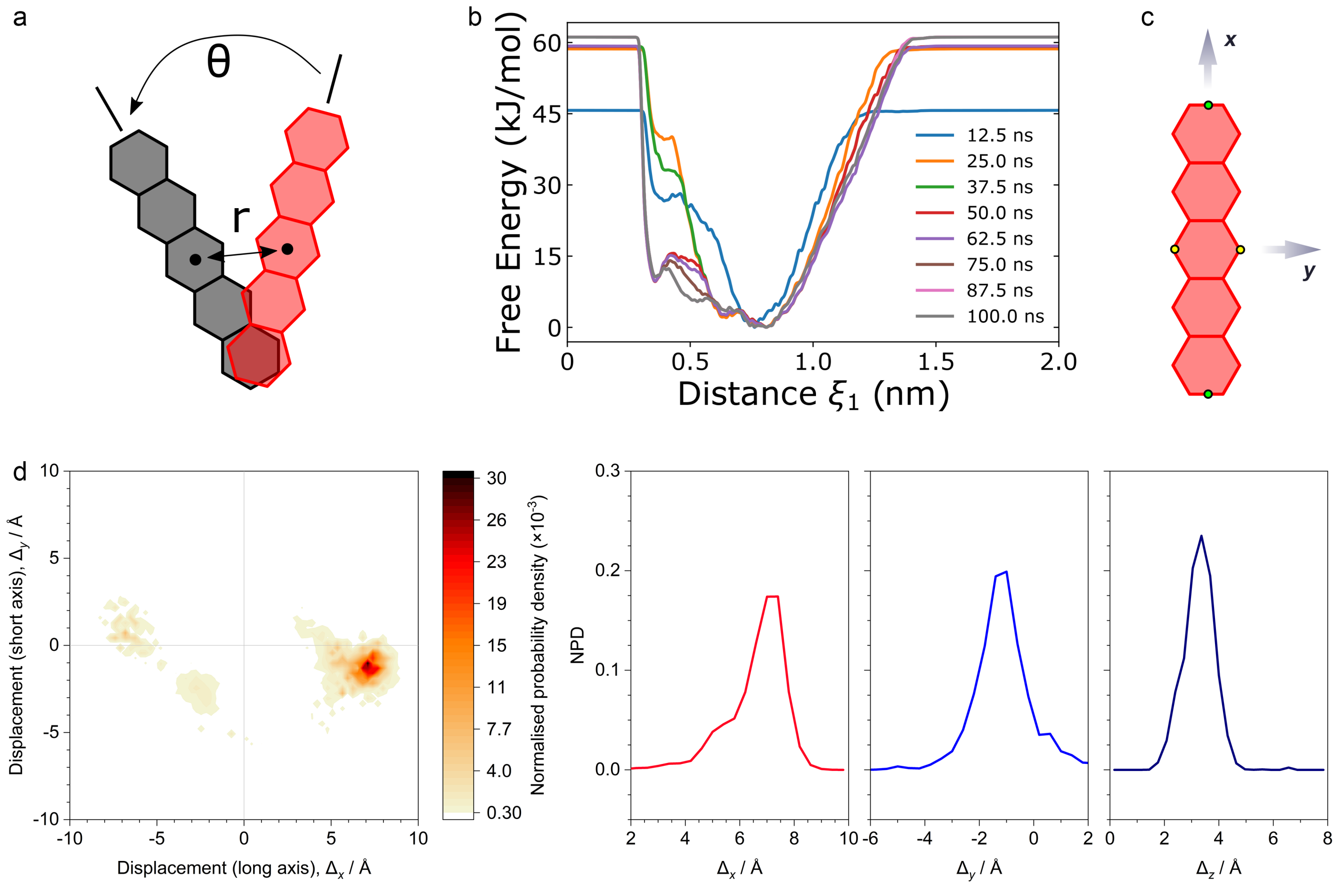

Figure S1. MD simulation setup and convergence.

(a) Illustration of the collective variables ξ_1 (*r*) and ξ_2 (θ) for the metadynamics simulations.

(b) Convergence of the metadynamics simulations assessed across simulation time assessed using the distance collective variable.

(c) Illustration of the points used to define the long (*x*, green dots) and short (*y*, yellow) molecular axes of the pentacene molecule.

(d) MD analysis of **PEN$_2$** (see Figure 2). Left: Normalised probability density (NPD) for displacements along the *x* and *y* molecular axes. Right: 1D representation of the NPD profiles for displacements along the *x*, *y*, and *z* molecular axes.

E. Self-assembled dimer (ssDNA-PEN)$_2$

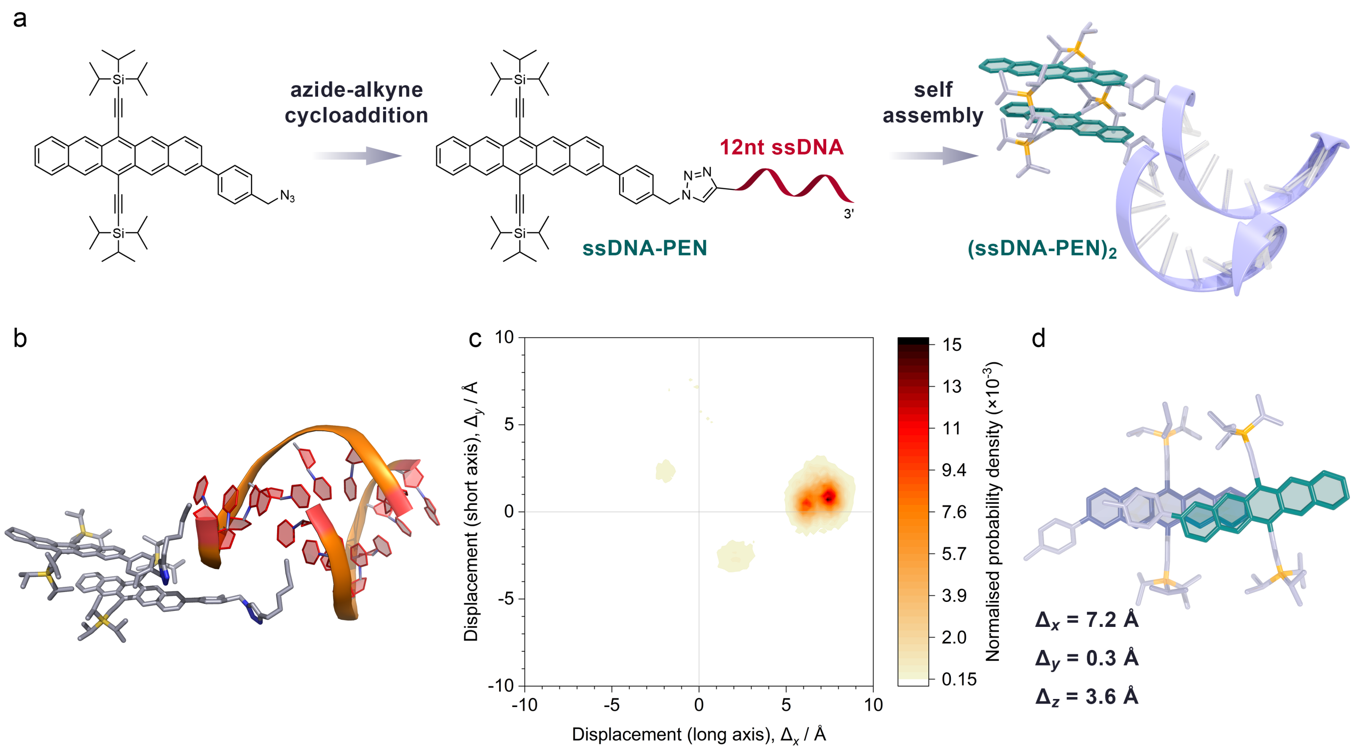

Figure S2. Synthesis and modelling of the self-assembled pentacene dimer.

(a) Pentacene is attached to ssDNA via Cu(I)-catalysed azide-alkyne cycloaddition. When dissolved in aqueous buffer (100 µM PBS), **ssDNA-PEN** self-assembles into dimers due to hydrophobic/hydrophilic interactions.

(b) Molecular dynamics (MD) simulations predict the self-assembly of **ssDNA-PEN** into **(ssDNA-PEN)$_2$** dimers. In the MD-optimised geometry, the two ssDNA strands associate into a weakly bound double-helix and the pentacenes form a slip-stacked dimer.

(c) MD analysis of the pentacene dimer geometries in **(ssDNA-PEN)$_2$**. The map shows the normalised probability density for the lateral offset along the pentacene long (Δ_x) and short (Δ_y) molecular axes. The simulation indicates a range of probable configurations with slightly more or less slip-stacked geometry, which are likely to co-exist in the samples.

(d) In the MD-optimised geometry, the pentacenes form slip-stacked cofacial dimers with a stacking distance (Δ_z) of 3.6 Å and large longitudinal offset of 7.2 Å.

Attachment of one ssDNA to the pentacene semiconductor yields a highly amphiphilic compound with the non-polar, strongly hydrophobic TIPS-pentacene on one side and the charged, hydrophilic ssDNA on the other (SI, Figure S2a). When dissolved in aqueous buffer solution, this **ssDNA-PEN** self-assembles spontaneously into dimers where the hydrophobic pentacenes aggregate to minimise their interface with the surrounding polar medium. This spontaneous dimer formation is fully reversible when a solvent that can dissolve the pentacene moieties is added. In DMSO/buffer 95:5 (*v*:*v*), **ssDNA-PEN** is monomeric (see SI, Figure S7).

To study this dimer formation and assess the dimer geometry, we employed molecular dynamics (MD) simulations, as described in the SI, Section D. Starting from two separated ssDNA-PEN, the simulation converges to a dimer, in which the pentacenes arrange in a slip-stacked cofacial geometry and the two identical, non-complementary ssDNA form a weakly bound double helix (SI, Figure S2b, S3a). Since both ssDNA strands have the same base sequence and directionality, their association is much weaker than in a DNA duplex of two complementary strands.

Interestingly, the self-assembly of **ssDNA-PEN** generates dimers selectively and does not extend towards larger aggregates. When we initialise the simulation with three **ssDNA-PEN**, only two of them dimerise, while the third pentacene remains well separated (SI, Figure S3b). This finding is supported by electron microscopy studies (SI, Section G), where we observe homogeneous samples with objects whose size is fully consistent with the simulated **(ssDNA-PEN)$_2$** dimers, but no indication of more extended structures.

While the hydrophobic/hydrophilic contrast is the main driving force for the dimer formation, controlling the dimer geometry on a sub-molecular length scale requires additional interactions (SI, Figure S4a). The pentacene core is a flat aromatic unit that favours cofacial arrangements with typical π-π distances of around 3.5 Å. The triisopropylsilyl (TIPS) ethynyl substituents on the 6 and 13 positions are sterically demanding but positioned with sufficient distance from the core to allow the π-stacking of adjacent pentacenes. Due to their steric bulk, they enforce a substantial offset of 6-8 Å along the long pentacene molecular axis, while simultaneously limiting the offset along the short molecular axis to below 1 Å. This way, the molecular geometry generates a docking site, which locks the second pentacene in a very well-defined geometry. The effect of this docking site leads to very similar dimer geometries in the DNA-linked **PEN$_2$** and the self-assembled **(ssDNA-PEN)$_2$**, which both closely resemble the packing motif in crystalline TIPS-pentacene (SI, Figure S4b).

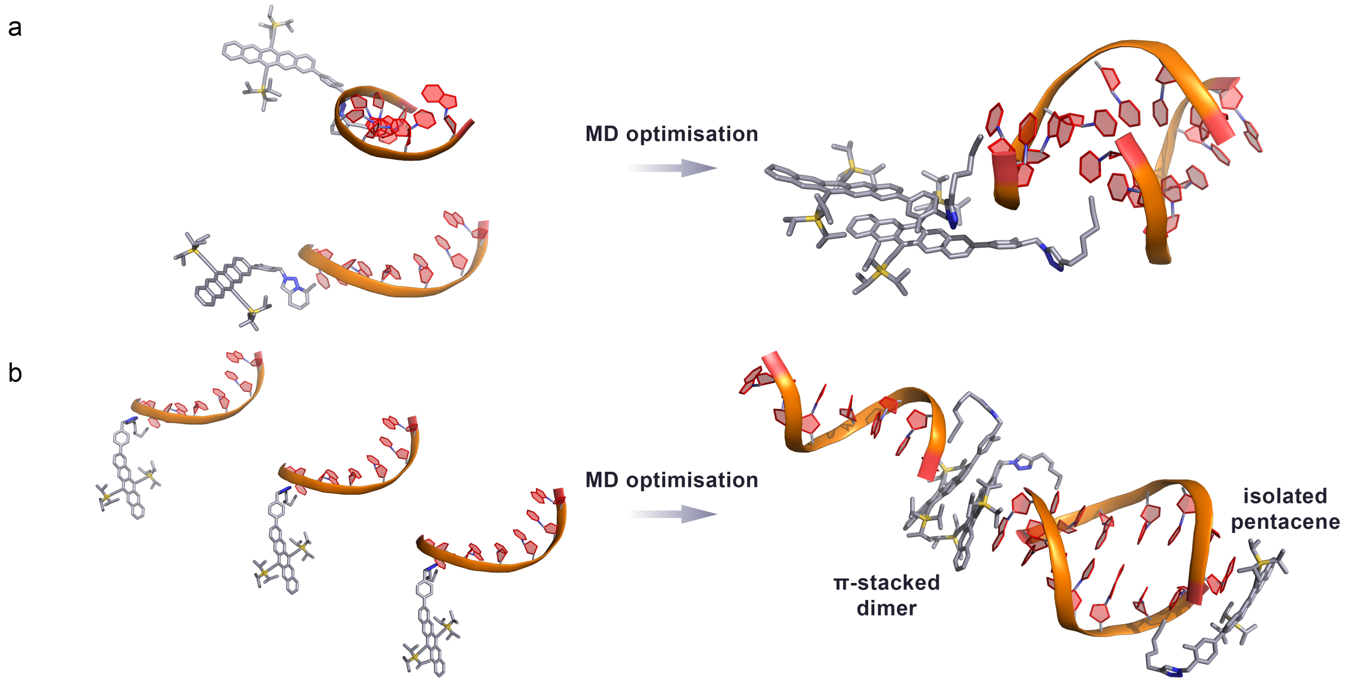

Figure S3. MD optimisation of **ssDNA-PEN** aggregates. (a) Initial (left) and optimised (right) geometries of the dimeric **(ssDNA-PEN)$_2$**. (b) MD optimisation of a hypothetical **ssDNA-PEN** trimer. We find that only two **ssDNA-PEN** dimerise, while the third one (bottom right) remains electronically isolated.

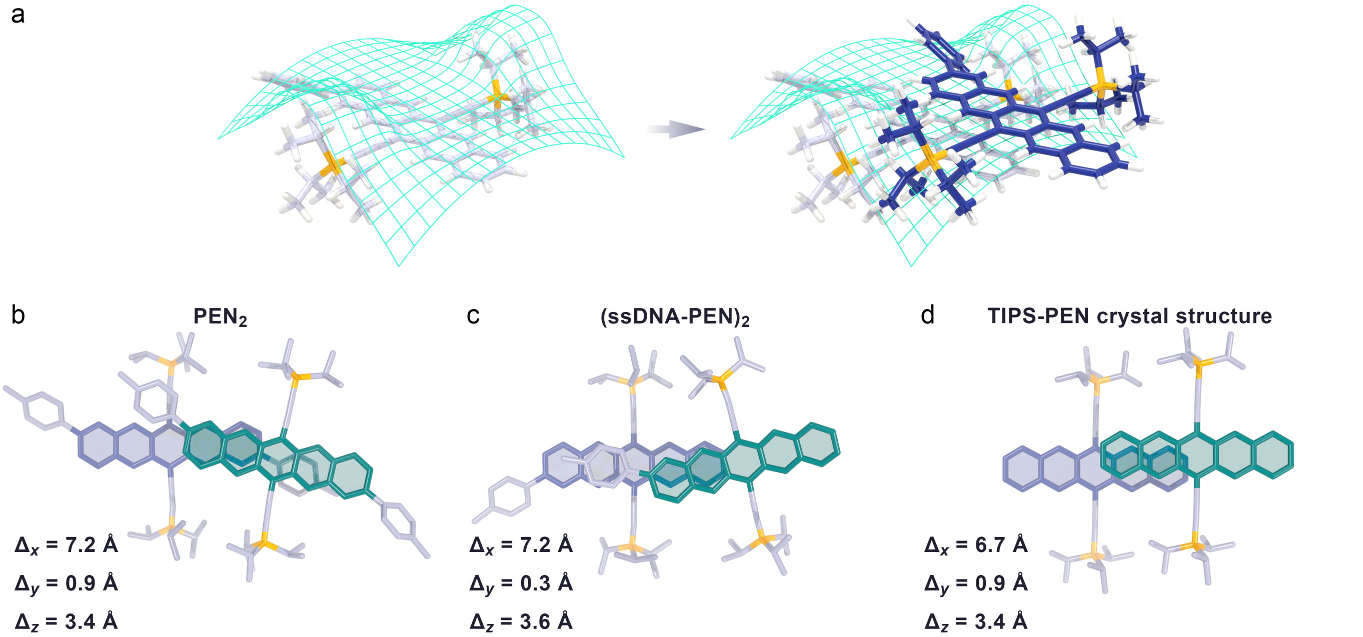

Figure S4. (a) The flat pentacene aromatic core with its two TIPS-ethynyl substituents generates a molecular docking site (illustrated in green), which guides a second pentacene (blue) into a very well-defined position. (b-d) This docking site causes very similar slip-stacked geometries in the DNA-linked **PEN$_2$** and the self-assembled **(ssDNA-PEN)$_2$**, which closely resemble the packing in TIPS-pentacene crystals.[29]

F. Modelling of larger constructs

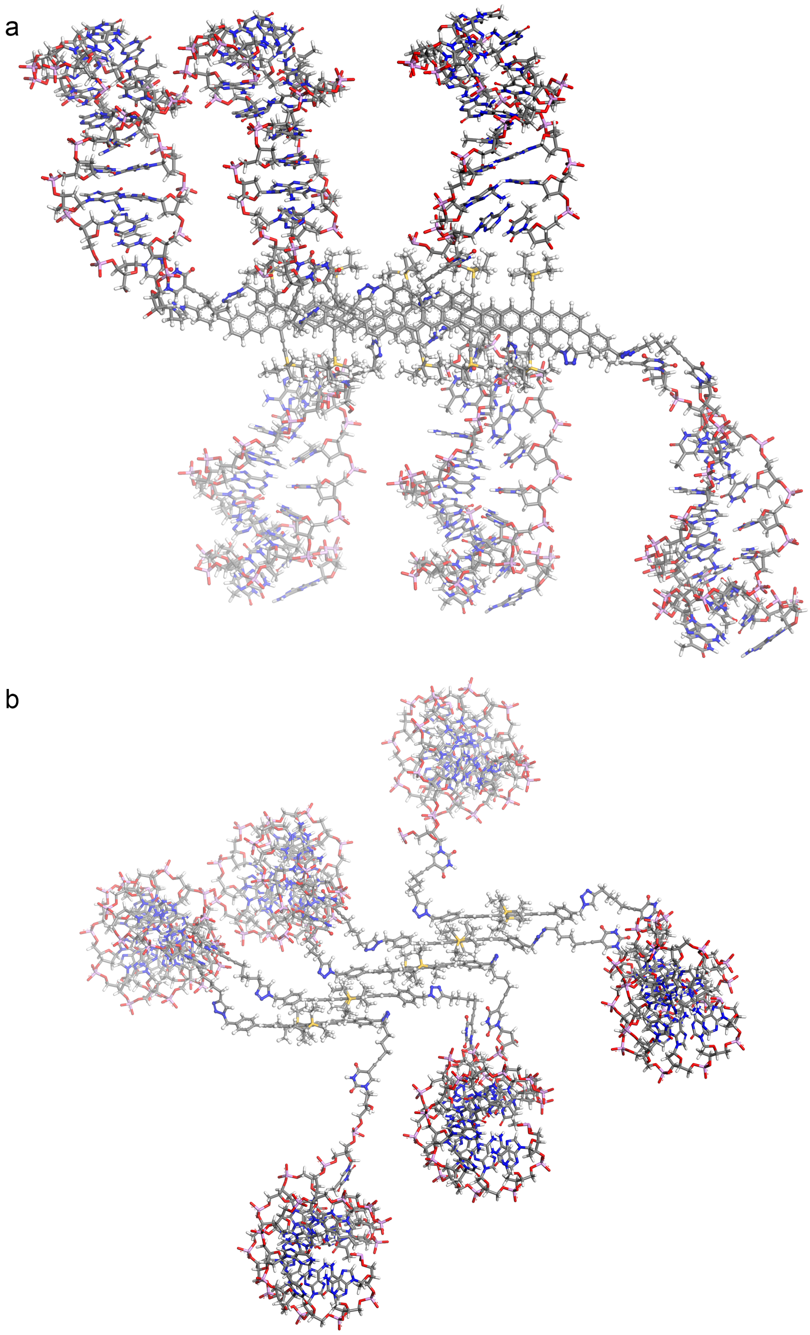

Figure S5. Force-field based modelling of **PEN$_5$**.

(a) Top view.

(b) Side view.

Due to the heavily offset packing of the pentacenes, there is sufficient space to accommodate the dsDNA on both sides of the pentacene stack.

G. Cryogenic transmission electron microscopy

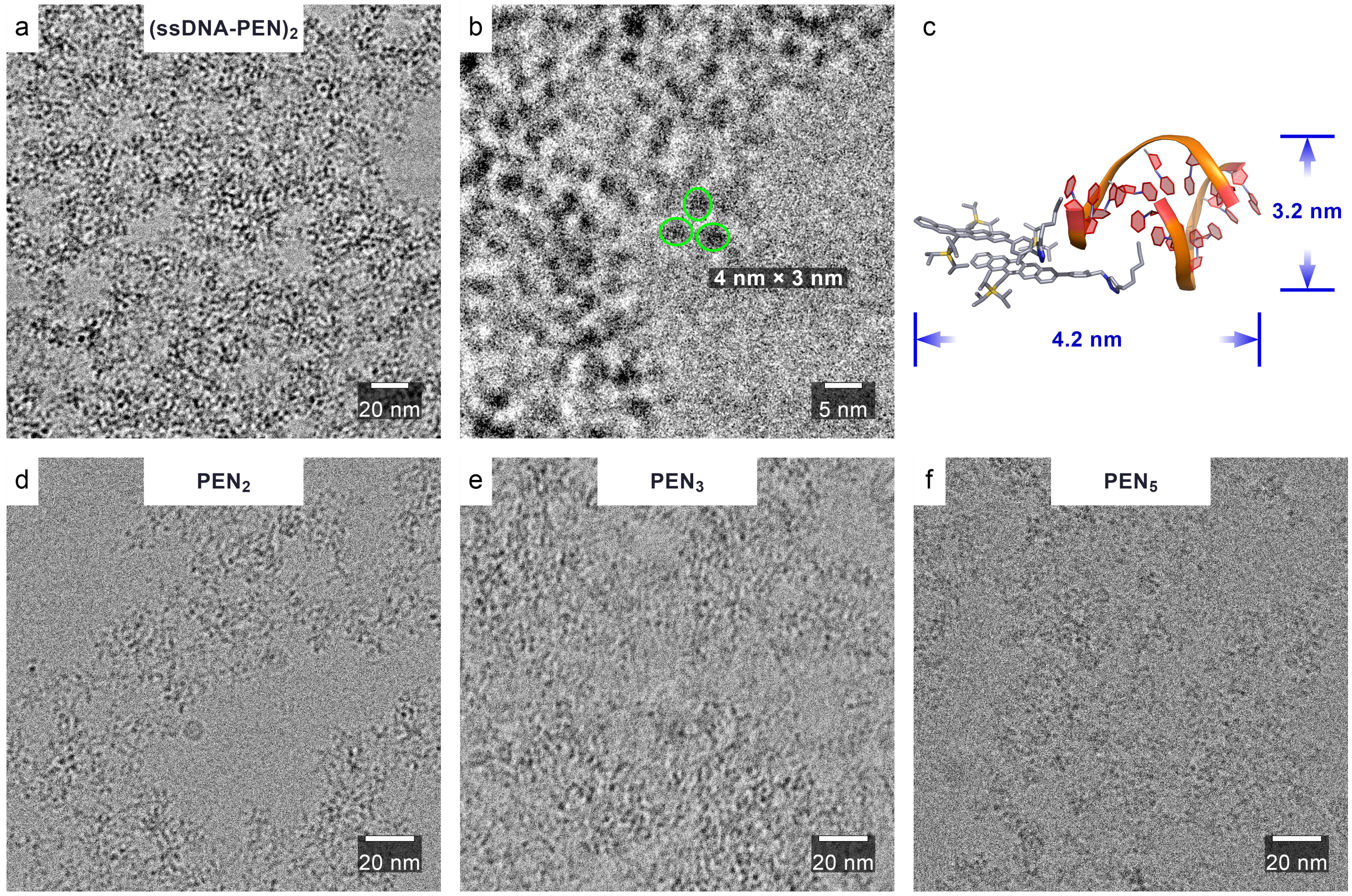

Figure S6. Cryogenic transmission electron microscopy (cryo-TEM) images of the pentacene constructs in vitreous buffer at 77 K.

(a-c) The self-assembled **(ssDNA-PEN)$_2$** appears as slightly elliptical objects that are about 4 nm long and 3 nm wide. This is in excellent agreement with the dimensions predicted by the MD simulations and confirms that the self-assembly exclusively generates dimers and no larger structures.

(d-f) Images of the DNA-linked **PEN$_2$**, **PEN$_3$** and **PEN$_5$**.

H. Transient absorption spectra of the self-assembled (ssDNA-PEN)$_2$

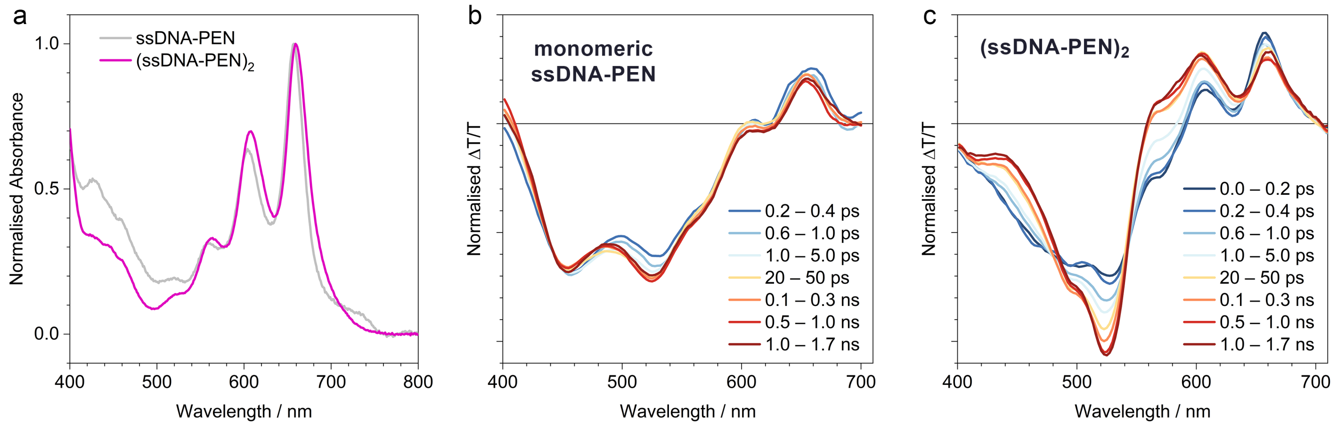

Figure S7. Spectroscopic characterisation of the self-assembled pentacene dimer. The monomeric **ssDNA-PEN** is dissolved in a DMSO/buffer mixture (95:5 v/v) to suppress aggregation. The aggregated **(ssDNA-PEN)$_2$** is dissolved in PBS buffer. Pentacene concentration is 100 µM.

(a) Steady-state absorption spectra of the self-assembled **(ssDNA-PEN)$_2$** and the monomeric **ssDNA-PEN**.

(b,c) fs-/ps TA spectra of the samples, following photoexcitation at 600 nm (pump fluence 5×10^{-5} J cm^{-2}). Spectra are normalised to their respective integrals.

Note: The signals of the monomeric **ssDNA-PEN** and the self-assembled **(ssDNA-PEN)$_2$** are sharper compared to the DNA-linked **PEN$_n$** series, because **ssDNA-PEN** contain only one isomer, whereas the **PEN$_n$** series contain a mixture of meta- and para-phenylene isomers (see SI, Section B).

I. Genetic algorithm deconvolution of the fs-/ps transient absorption spectra

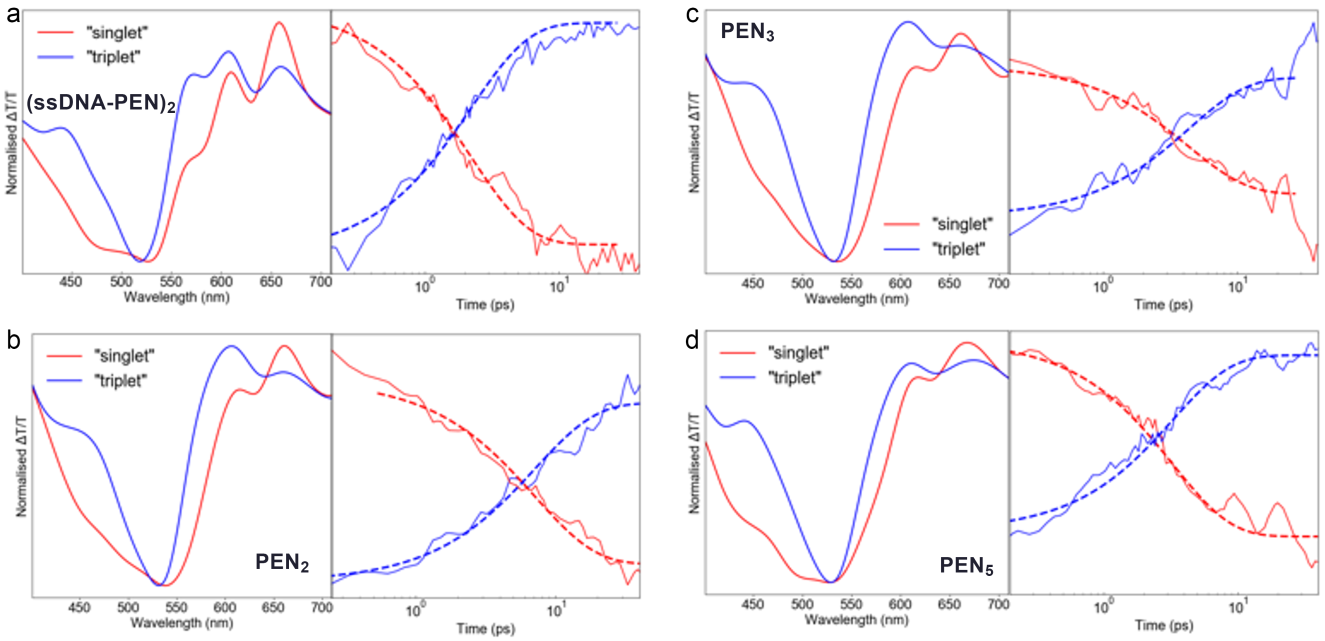

Figure S8. Species associated spectra (left panel) and kinetics (right panel), attributed to singlet (red) and triplet (blue) excited state species, outputted from GA of the fs-/ps-TA spectra of (a) **(ssDNA-PEN)$_2$**, (b) **PEN$_2$**, (c) **PEN$_3$** and (d) **PEN$_5$**. The corresponding TA spectra are shown in Figure 2 (DNA-linked assemblies) and SI, Figure S7 (self-assembled dimer), respectively. Overlaid on the kinetic plots are the single exponential kinetic fits (dashed lines), the parameters of which are summarised in the table below. The outputted singlet fission time constants, τ_{SF}, are 2.2, 7.1, 4.4 and 3.3 ps for (a) – (d) respectively.

Deconvolution of the spectra allows us to extract the fission rates of the DNA assemblies. We use a genetic algorithm (GA) to obtain species associated spectra and kinetics.[30] We use the characteristic absorptive feature at 450 nm, present in the pentacene singlet spectra of the monomeric **ssDNA-PEN** (Figure 2b and SI, Figure S7b) and isolated from absorptive triplet features, as an indicator of the singlet associated species. The other spectra, which exhibit a strong absorptive feature at 530 nm, are assigned to the triplet species (SI, Figure S8, left panels). We subsequently fit the kinetic decays and growths of the singlet and triplet associated species respectively to single exponential functions (SI, Figure S8, right panels), such that the rate of triplet growth matches that of singlet decay, using the following function:

$$\frac{\Delta T}{T} = Ae^{-(t+B)/\tau} + C$$

The outputted parameters are summarised in the table below:

	Sample	τ_{SF} / ps	A	B	C
**PEN$_2$**	"singlet"	7.1	2.44	46.6	-1.00
	"triplet"	7.1	-1.11×10^{-2}	8.36	1.00
**PEN$_3$**	"singlet"	4.4	9.55×10^{-1}	1.31	2.45×10^{-1}
	"triplet"	4.4	-1.02	1.27	8.83×10^{-1}
**PEN$_5$**	"singlet"	3.3	3.10×10^{-1}	-3.99	-9.14×10^{-3}
	"triplet"	3.3	-5.54×10^{-1}	-1.72	9.71×10^{-1}
**(ssDNA-PEN)$_2$**	"singlet"	2.2	1.77×10^{-3}	1.74×10^{-1}	1.98×10^{-4}
	"triplet"	2.2	-3.20×10^{-1}	11.2	2.19×10^{-3}

J. Nanosecond transient absorption spectroscopy

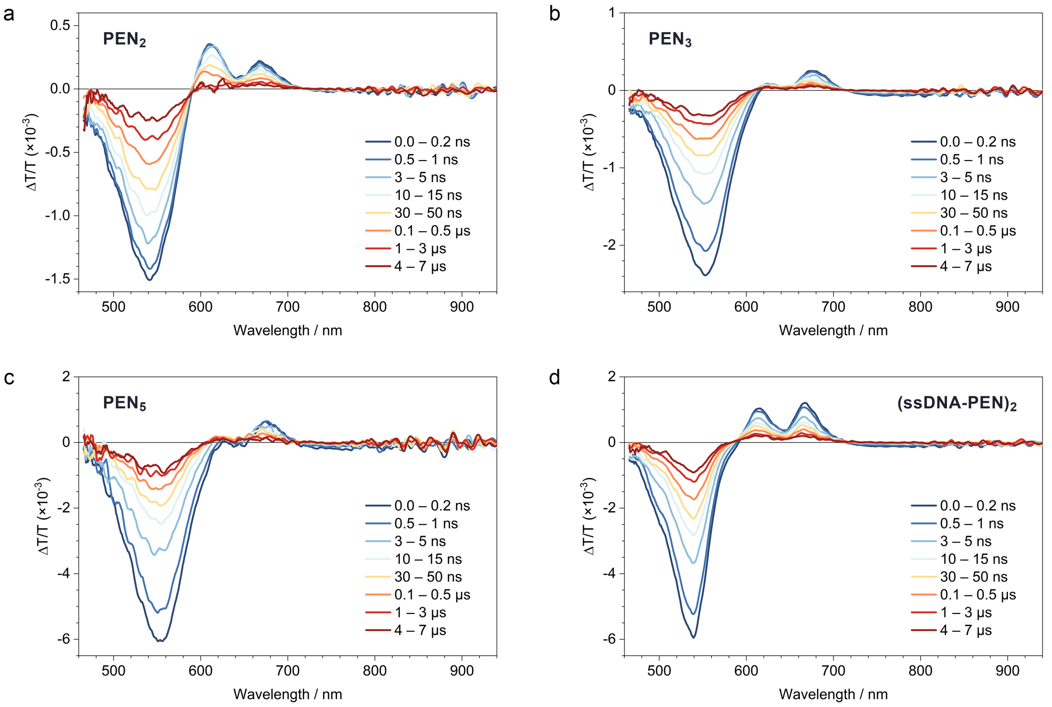

Figure S9. Nano-/microsecond TA spectra of (a) **PEN$_2$**, (b) **PEN$_3$**, (c) **PEN$_5$**, and (d) **(ssDNA-PEN)$_2$**. Samples are dissolved in PBS at 100 µM pentacene concentration. The measurements were taken at 400 nm photoexcitation (600 nm for **PEN$_2$**).

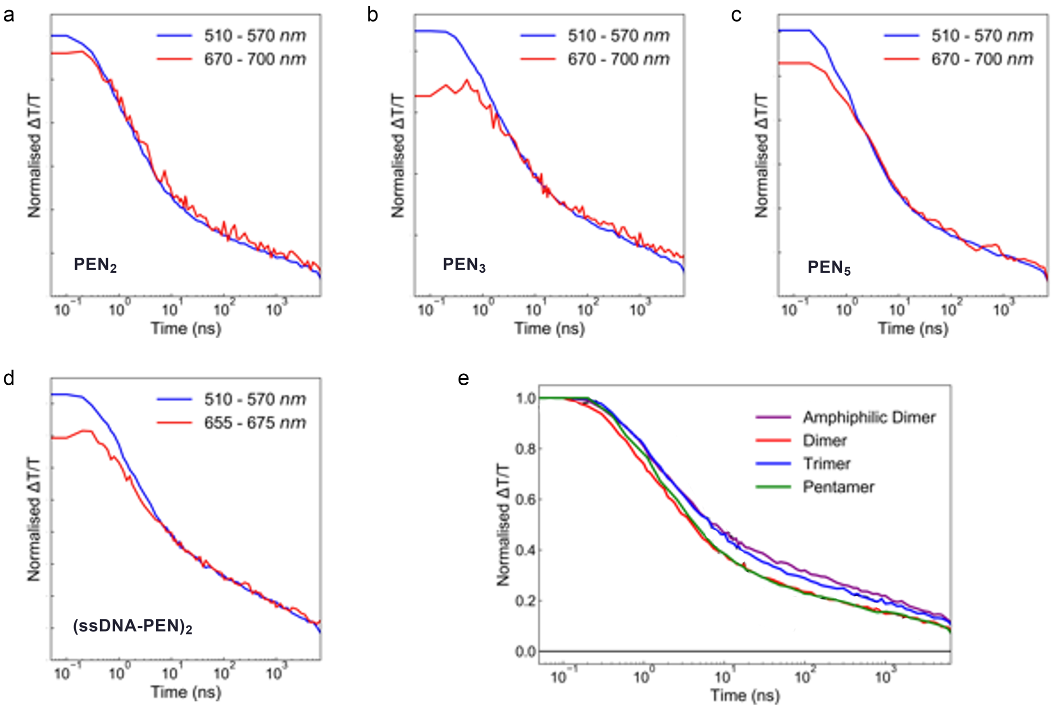

Figure S10. Kinetic decay profiles.

(a-d) Kinetic decay profiles of the triplet PIA (blue) and GSB (red) signals extracted from the ns/µs TA spectra shown in SI, Figure S9. Decay traces are normalised to the tail end at 10 ns.

(e) Comparison of the PIA decay profiles. The triplet species decays slightly slower in **PEN$_3$** and **(ssDNA-PEN)$_2$**. Decay traces are normalised to the maximum ΔT/T.

K. Transient ESR spectroscopy

Time-evolution of ESR spectra

Here we show the full transient ESR data, the intensity of microwave emission and absorption as a function of magnetic field and time after the laser flash, for each sample. The DNA-assembled pentacene samples (SI Figure S11, columns 1-3 from left) show the evolution from predominantly quintet to triplet through time, while the TIPS-pentacene film shows the evolution from the initial triplet to secondary triplet spectrum with an additional central feature consistent with charges or charge-transfer states (SI Figure S11, column 4).

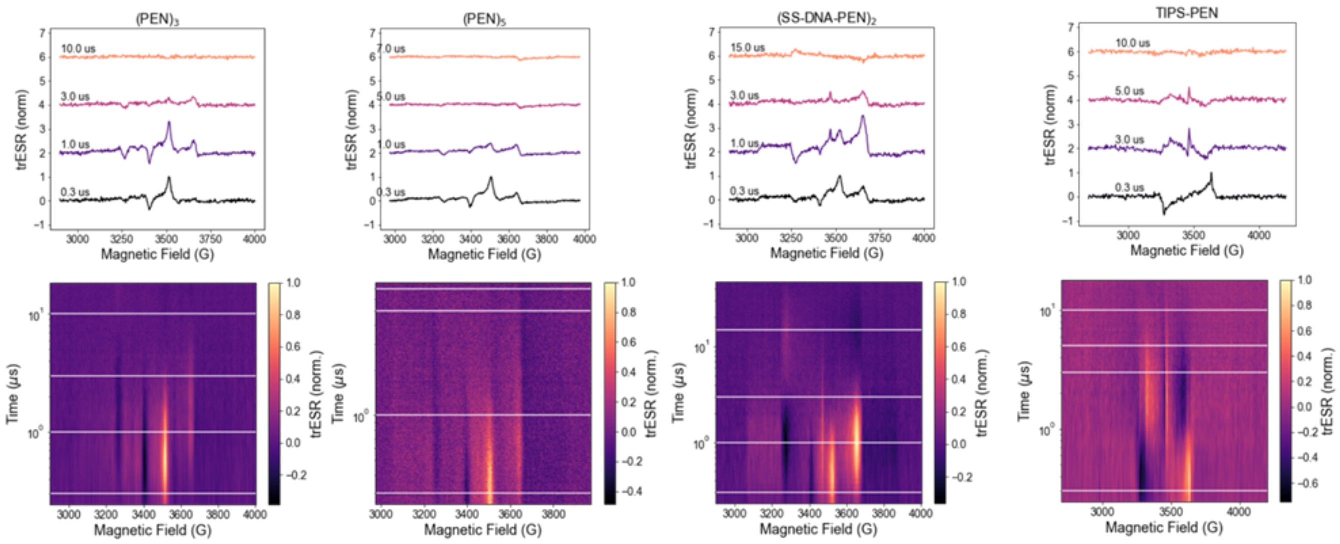

Figure S11. Spectral slices (top) and full trESR map (bottom) with time points marked in white for each sample labelled as in the main text. Temperature for all DNA assemblies is 50 K, while temperature for **TIPS-PEN** is 10 K.

We then take the quintet and triplet absorptive peak positions and plot the magnitude of the trESR signal as a function of time to extract a triplet and quintet lifetime. We note that the **TIPS-PEN** sample exhibits a rapid decay as the spectrum evolves into that shown at late times in SI Figure S11 (right). The fitted rise and decay times are given in SI Table S2 and all triplet states in the DNA-assembled samples are found to decay with a timescale of 1-2 μs.

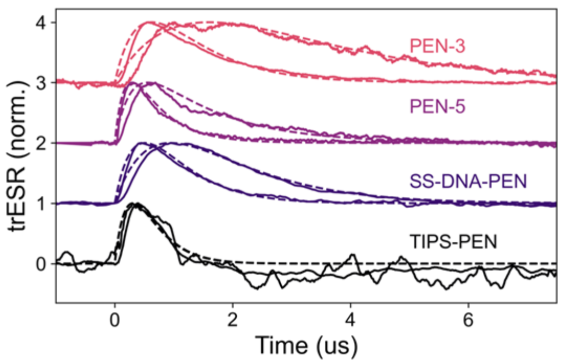

Figure S12. Kinetics for triplet and quintet absorptive peak transitions with dashed fitted lines and fit parameters given in Table S2 below.

Table S2. Exponential rise and decay time constants for triplet and quintet transitions marked at fields absorptive transition peaks and shown as kinetic slices in SI Figure S12.

Compound	Quintet rise time (µs)	Quintet decay time (µs)	Triplet rise time (µs)	Triplet decay time (µs)
**PEN_3**	0.5	0.8	1.6	1.6
**PEN_5**	0.17	0.5	0.3	1.2
**$(ssDNA\text{-}PEN)_2$**	0.5	0.6	1.0	1.0
**TIPS-PEN**	-/-	-/-	0.3	0.3

Least-squares fitting parameters of trESR spectra

Quintet spectra are taken from the earliest time species for each DNA assembled structure. For **$(ssDNA\text{-}PEN)_2$** we observe significant spectral overlap between triplet and quintet throughout the early time evolution, and so we separate independent spectral components to extract the early time quintet spectrum based on its distinct time-evolution using the independent component analysis (ICA) function in scikit-learn library.[31] The only DNA assembled structure with a clear, measured long lived triplet signal with no quintet component is the **$(ssDNA\text{-}PEN)_2$** sample. We therefore use this long-time spectrum to estimate the zero-field splitting (ZFS) parameters, i.e., spin energy level splitting in the absence of a magnetic field, of the triplet on the DNA-assembled pentacene derivatives. We fit these spectra for each sample by combining the open-source Matlab-based ESR library Easyspin[2] and least-squares linear regression in SciPy.[32]

The fitted D and E parameters define the ZFS parameters in MHz. The line broadening is taken to be Lorentzian in form and is reported as the full-width-half-maximum (FWHM) in Gauss.

The normalized spin-sublevel populations used to fit the spectra are further reported as follows. For S = 1 species the populations are either the high field populations of the m = 0,±1 sublevels quantized along the external magnetic field direction or the zero-field x,y,z populations where the x,y,z states are the eigenstates at zero magnetic field (indicated with an asterisk in Table S3 for **$(ssDNA\text{-}PEN)_2$**). For S = 2 species, populations are reported as the m = 0,±1,±2 eigenstates quantized along the external magnetic field direction. The ordering in Table S3 is from lowest to highest energy eigenstates.

Where necessary an orientational ordering parameter is used to capture a degree of selectivity in spin polarization with orientation of the molecular axes relative to the external magnetic field. This parameter is defined in the Easyspin documentation.[2] We note that we expect spin populations may be orientationally selective due to the dipolar contribution to spin mixing between the singlet and quintet sublevels. The negative fitted orientational ordering parameter indicates a slight preferential ordering of the xy axis to the B_0.

The best-fit parameters are summarized in the chart below with corresponding comparison to experimental spectra in SI Figure S13.

Table S3. Least-squares fitted spin parameters. All spin populations are high-magnetic field Zeeman energy levels ordered energetically unless subscripted x,y,z, which indicates zero-field triplet eigenstate populations.

Sample	Spin Multiplicity	D (MHz)	E (MHz)	Linewidth (G)	Spin-sublevel populations	Orientational Ordering Parameter	Experimental Time-slice (µs)
**TIPS-PEN**	1(early-time)<br>1(late-time)	1053±2<br>853±9	17±1<br>42±4	1.0±0.5<br>1.0±0.5	(0.0, 1.0, 0.0)<br>(0.5, 0.0, 0.5)	-0.8±0.1<br>N/A	0.2-0.35<br>4.0-6.0
**PEN_3**	2	349±3	15±2	1.0±0.2	(0.3 , 0.2, 0.4, 0.1, 0.0)	-0.3±0.1	0.20-0.35
**PEN_5**	2	351±5	11±3	1.0±0.2	(0.27, 0.26, 0.39, 0.08 , 0.0)	-0.5±0.1	0.05-0.15
**$(ssDNA\text{-}PEN)_2$**	2<br>1	350±3<br>1108±4	10±2<br>16±2	1.2±0.2<br>0.6±0.1	(0.24, 0.26, 0.4 , 0.06, 0.04)<br>(0.0,0.0,1.0)x,y,z	-0.9±0.1<br>N/A	Extr. using ICA[31]<br>10.0-20.0

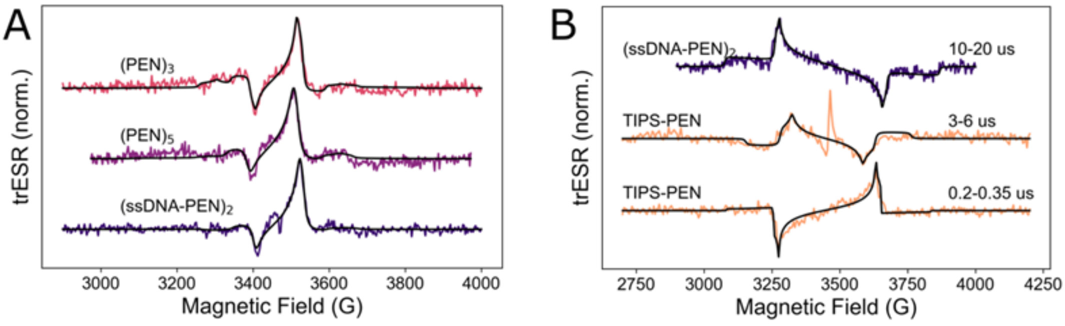

Figure S13. Fitted S = 2 quintet (**a**) and S = 1 triplet (**b**) spectra in black overlayed with corresponding experimental data from parameters in SI Table S3. Experimental time points for each spectrum are listed in SI Table S3 above.

Theoretical quintet (S=2) zero-field splitting parameters

We compare the fitted ZFS parameters D_q^{exp}, E_q^{exp} to the theoretical ZFS parameters taken from the geometry of the triplet pairs given by molecular dynamics simulations described in SI Section D. The theoretical quintet ZFS parameters from molecular dynamics ($D_q^{theory}, E_q^{theory}$) are determined following the theory laid out in Ref [33]. In short, in the limit of strong exchange coupling between triplets ($J \gg D_T$) where J is the inter-triplet exchange interaction and D_T is the intratriplet dipolar interaction strength. The quintet zero field splitting is determined by the combination of intratriplet dipolar interactions and intertriplet dipolar interactions as follows.

The quintet zero-field splitting Hamiltonian $\frac{\hat{H}_{zfs}}{h} = \boldsymbol{S^T} \cdot \boldsymbol{D}_q \cdot \boldsymbol{S} = D_q(\hat{S}_z^2 - 2\hat{I}_3) + E_q(\hat{S}_z^2 - \hat{S}_z^2)$ where $\boldsymbol{S}$ is the vector of spin operators, $\hat{I}_3$ is the three-dimensional identity matrix, $\hat{S}_i$ is the spin operator defined along the principal axes $i \in (\hat{x}_q, \hat{y}_q, \hat{z}_q)$ of the ZFS-tensor $\boldsymbol{D}_q$, determined by the symmetry of the underlying spin-spin interactions. The quintet ZFS tensor $\boldsymbol{D}_q$ can be written in terms of the ZFS parameters of each underlying triplet and the dipolar interactions between triplets (here labelled as the triplet on molecule a and molecule b) as $\boldsymbol{D}_q = \frac{\boldsymbol{D_T}}{\boldsymbol{6}}\left(\sum_{i=a,b} \hat{z}_i \hat{z}_i^T - \frac{2}{3}\hat{I}_3\right) + \frac{E_T}{6}\left(\sum_{i=a,b} \hat{x}_i \hat{x}_i^T - \hat{y}_i \hat{y}_i^T\right) - \frac{\Gamma}{3}(\hat{u}_{ab}\hat{u}_{ab}^T - \frac{1}{3}\hat{I}_3)$ where D_T and E_T are the triplet ZFS parameters, $(\hat{x}_i, \hat{y}_i, \hat{z}_i)$, $i \in a, b$ are the symmetry axes for the triplets on molecule a and b, $\Gamma = 3\mu_0\mu_B^2 g^2/4\pi|\vec{r}_{ab}|^3$ is the strength of the inter-triplet dipolar interaction where μ_0 is the magnetic permeability of free space, μ_B the Bohr magneton, g the g-factor, $\vec{r}_{ab}$ the center-to-center vector between triplet-bearing molecules, and $\hat{u}_{ab}$ the unit vector between molecules a and b. We note that a more detailed derivation is given in Ref [33].

The input parameters we use are $D_T, E_T = (1108{,}16)$ MHz (see section above) and $\hat{x}_i, \hat{y}_i, \hat{z}_i$ axes defined along the long, short, and out-of-plane axes.[34] The relative orientations of each set of triplet axes and the inter-triplet dipolar interaction strength and unit vector we then take from molecular dynamics calculated geometry (see SI Section D for MD simulation details). In SI Table S4, we report the resulting theoretical quintet ZFS parameter ($D_q^{theory}, E_q^{theory}$) along with the inter-triplet center-to-center distance.

Table S4. Calculated theoretical ZFS values for the experimental crystal structure of TIPS-pentacene, the MD-simulated dimer structure of DNA-linked TIPS-pentacene, and the fitted values from ESR described above.

	$\boldsymbol{D_q^{theory}}$ (MHz)	$\boldsymbol{E_q^{theory}}$ (MHz)	$\lvert\vec{\boldsymbol{r}}_{\boldsymbol{ab}}\rvert$ (Å)
**TIPS-PEN***	400	46	7.6
MD Geometry†	390	37	7.9
Measured (ESR)	350(5)	12(5)	-/-

*Geometry from reported crystal structure[29], †Geometry described in Figure 1 and SI Section D,E.

The systematic offset in the measured D_q, E_q parameters relative to the theoretical predictions could be due to strain in the dimer geometries in experiments versus theory. To determine whether this is possible, we simulate the degree to which the measured ZFS parameters vary with variation in intermolecular geometry using reasonable possible distortions. Namely we allow the inter-triplet distance to vary following a normal distribution of each component of $\vec{r}_{ab} = (r_x, r_y, r_z)$ with standard deviation of 0.5 Å and variation in the rotation of each triplet ZFS axes with a standard deviation of 10° (rotation about a uniformly distributed random axis). We then compare the distribution of $D_q^{theory}, E_q^{theory}$ to the measured values with least-squares fitted ZFS parameters shown in black and standard error from fitted spectra in grey. As shown in SI Figure S14 the experimentally measured D_q, E_q values fall within the distribution of $D_q^{theory}, E_q^{theory}$ values from MD simulations including a random degree of strain. We note that D_q, E_q are sensitive to both (1) inter-triplet distance and (2) relative rotation of the pentacene cores and both factors likely contribute together to the observed systematic shift in measured D_q, E_q relative to the MD simulation. We also note that a pair residing on next-nearest neighbors with $J > D$ would have reduced ZFS parameter values and this could also contribute to the distinct values compared to theory.

The function from intermolecular geometry to ZFS parameters is not one-to-one and so it is not possible to extract a single geometry from the measured *D*,*E* parameters. We conclude that while the measured ZFS parameters are not consistent with the exact intermolecular geometry given by MD simulations, they would be consistent with a small degree of strain in frozen solution compared to in silico molecular dynamics.

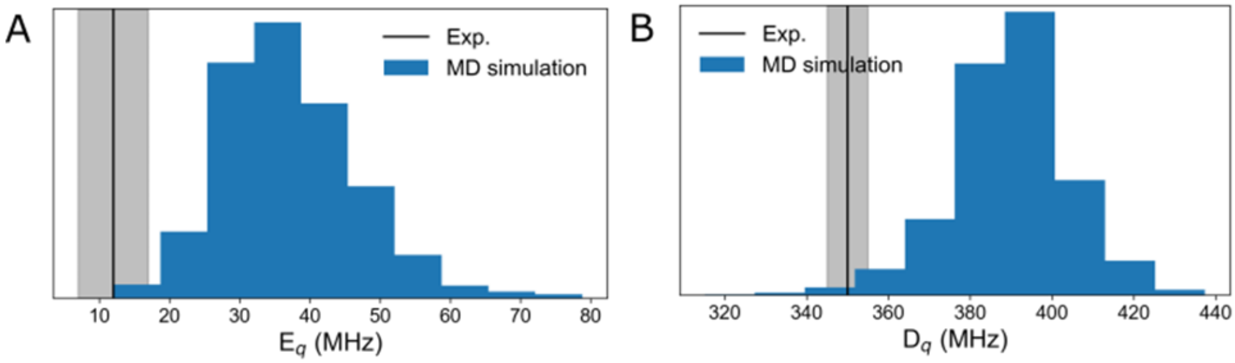

Figure S14. Comparison between the experimental (black) and calculated distribution (blue) of the *D*,*E* parameters.

L. References

[1] J. C. de Mello, H. F. Wittmann, R. H. Friend, *Adv. Mater.* **1997**, *9*, 230.
[2] S. Stoll, A. Schweiger, *J. Magn. Reson.* **2006**, *178*, 42.
[3] A. J. Tilley, R. D. Pensack, E. L. Kynaston, G. D. Scholes, D. S. Seferos, *Chem. Mater.* **2018**, *30*, 4409.
[4] E. Kumarasamy, S. N. Sanders, A. B. Pun, S. A. Vaselabadi, J. Z. Low, M. Y. Sfeir, M. L. Steigerwald, G. E. Stein, L. M. Campos, *Macromolecules* **2016**, *49*, 1279.
[5] E. F. Pettersen, T. D. Goddard, C. C. Huang, G. S. Couch, D. M. Greenblatt, E. C. Meng, T. E. Ferrin, *J. Comput. Chem.* **2004**, *25*, 1605.
[6] E. Vanquelef, S. Simon, G. Marquant, E. Garcia, G. Klimerak, J. C. Delepine, P. Cieplak, F.-Y. Dupradeau, *Nucleic Acids Res.* **2011**, W511.
[7] C. I. Bayly, P. Cieplak, W. Cornell, P. A. Kollman, *J. Phys. Chem.* **1993**, *97*, 10269.
[8] J. Wang, W. Wang, P. A. Kollman, D. A. Case, *J. Mol. Graph. Model.* **2006**, *25*, 247.
[9] J. Wang, R. M. Wolf, J. W. Caldwell, P. A. Kollman, D. A. Case, *J. Comput. Chem.* **2004**, *25*, 1157.
[10] X. Dong, X. Yuan, Z. Song, Q. Wang, *Phys. Chem. Chem. Phys.* **2021**, *23*, 12582.
[11] M. D. Hanwell, D. E. Curtis, D. C. Lonie, T. Vandermeersch, E. Zurek, G. R. Hutchison, *J. Cheminform.* **2012**, *4*, 17.
[12] D. A. Case, T. E. Cheatham III, T. Darden, H. Gohlke, R. Luo, K. M. Merz Jr., A. Onufriev, C. Simmerling, B. Wang, R. J. Woods, *J. Comput. Chem.* **2005**, *26*, 1668.
[13] I. Ivani, P. D. Dans, A. Noy, A. Pérez, I. Faustino, A. Hospital, J. Walther, P. Andrio, R. Goñi, A. Balaceanu, G. Portella, F. Battistini, J. L. Gelpí, C. González, M. Vendruscolo, C. A. Laughton, S. A. Harris, D. A. Case, M. Orozco, *Nat. Methods* **2016**, *13*, 55.
[14] W. L. Jorgensen, J. Chandrasekhar, J. D. Madura, *J. Chem. Phys.* **1983**, *79*, 926.
[15] I. S. Joung, T. E. Cheatham, *J. Phys. Chem. B* **2008**, *112*, 9020.
[16] M. J. Abraham, T. Murtola, R. Schulz, S. Páll, J. C. Smith, B. Hess, E. Lindahl, *SoftwareX* **2015**, *1*, 19.
[17] M. R. Shirts, C. Klein, J. M. Swails, J. Yin, M. K. Gilson, D. L. Mobley, D. A. Case, E. D. Zhong, *J. Comput. Aided Mol. Des.* **2017**, *31*, 147.
[18] G. Bussi, D. Donadio, M. Parrinello, *J. Chem. Phys.* **2007**, *126*, 014101.
[19] M. Parrinello, *J. Appl. Phys.* **1981**, *52*, 7182.
[20] T. Darden, D. York, L. Pedersen, *J. Chem. Phys.* **1993**, *98*, 10089.
[21] U. Essmann, L. Perera, M. L. Berkowitz, T. Darden, H. Lee, L. G. Pedersen, *J. Chem. Phys.* **1995**, *103*, 8577.
[22] M. Bonomi, D. Branduardi, G. Bussi, C. Camilloni, D. Provasi, P. Raiteri, D. Donadio, F. Marinelli, F. Pietrucci, R. A. Broglia, M. Parrinello, *Comput. Phys. Commun.* **2009**, *180*, 1961.
[23] G. A. Tribello, M. Bonomi, D. Branduardi, C. Camilloni, G. Bussi, *Comput. Phys. Commun.* **2014**, *185*, 604.
[24] N. Michaud-Agrawal, E. J. Denning, T. B. Woolf, O. Beckstein, *J. Comput. Chem.* **2011**, *32*, 2319.
[25] A. Laio, M. Parrinello, *Proc. Natl. Acad. Sci. U. S. A.* **2002**, *99*, 12562.
[26] A. Barducci, G. Bussi, M. Parrinello, *Phys. Rev. Lett.* **2008**, *100*, 020603.
[27] J. Gorman, S. R. E. Orsborne, A. Sridhar, R. Pandya, P. Budden, A. Ohmann, N. A. Panjwani, Y. Liu, J. L. Greenfield, S. Dowland, V. Gray, S. T. J. Ryan, S. De Ornellas, A. H. El-Sagheer, T. Brown, J. R. Nitschke, J. Behrends, U. F. Keyser, A. Rao, R. Collepardo-Guevara, E. Stulz, R. H. Friend, F. Auras, *J. Am. Chem. Soc.* **2022**, *144*, 368.
[28] M. Bonomi, A. Barducci, M. Parrinello, *J. Comput. Chem.* **2009**, *30*, 1615.
[29] J. E. Anthony, J. S. Brooks, D. L. Eaton, S. R. Parkin, *J. Am. Chem. Soc.* **2001**, *123*, 9482.
[30] S. Gélinas, O. Paré-Labrosse, C.-N. Brosseau, S. Albert-Seifried, C. R. McNeill, K. R. Kirov, I. A. Howard, R. Leonelli, R. H. Friend, C. Silva, *J. Phys. Chem. C* **2011**, *115*, 7114.
[31] F. Pedregosa, G. Varoquaux, A. Gramfort, V. Michel, B. Thirion, O. Grisel, M. Blondel, P. Prettenhofer, R. Weiss, V. Dubourg, J. Vanderplas, A. Passos, D. Cournapeau, M. Brucher, M. Perrot, É. Duchesnay, *J. Machine Learning Res.* **2011**, *12*, 2825.
[32] P. Virtanen, R. Gommers, T. E. Oliphant, M. Haberland, T. Reddy, D. Cournapeau, E. Burovski, P. Peterson, W. Weckesser, J. Bright, S. J. van der Walt, M. Brett, J. Wilson, K. J. Millman, N. Mayorov, A. R. J. Nelson, E. Jones, R. Kern, E. Larson, C. J. Carey, İ. Polat, Y. Feng, E. W. Moore, J. Van der Plas, D. Laxalde, J. Perktold, R. Cimrman, I. Henriksen, E. A. Quintero, C. R. Harris, A. M. Archibald, A. H. Ribeiro, F. Pedregosa, P. van Mulbregt, S. Contributors, *Nat. Methods* **2020**, *17*, 261.
[33] K. M. Yunusova, S. L. Bayliss, T. Chanelière, V. Derkach, J. E. Anthony, A. D. Chepelianskii, L. R. Weiss, *Phys. Rev. Lett.* **2020**, *125*, 097402.
[34] T. Yago, G. Link, G. Kothe, T.-S. Lin, *J. Chem. Phys.* **2007**, *127*, 114503.
